# SIRT2 Promotes HBV Transcription and Replication by Targeting Transcription Factor p53 to Increase the Activities of HBV Enhancers and Promoters

**DOI:** 10.3389/fmicb.2022.836446

**Published:** 2022-05-19

**Authors:** Dai-Qing Wu, Qiu-Ying Ding, Na-Na Tao, Ming Tan, Yuan Zhang, Fan Li, Yu-Jiao Zhou, Mei-Ling Dong, Sheng-Tao Cheng, Fang Ren, Juan Chen, Ji-Hua Ren

**Affiliations:** ^1^The Key Laboratory of Molecular Biology of Infectious Diseases (Chinese Ministry of Education), Chongqing Medical University, Chongqing, China; ^2^Key Laboratory of Molecular Biology for Infectious Diseases, Centre for Lipid Research, Ministry of Education, Chongqing Medical University, Chongqing, China; ^3^Department of Clinical Laboratory, Chongqing Traditional Chinese Medicine Hospital, Chongqing, China; ^4^Department of Endocrine and Breast Surgery, The First Affiliated Hospital of Chongqing Medical University, Chongqing, China

**Keywords:** hepatitis B virus, SIRT2, p53, transcription, cccDNA

## Abstract

Chronic hepatitis B (CHB) virus infection is one of the leading causes of cirrhosis and liver cancer. Although the major drugs against CHB including nucleos(t)ide analogs and PEG-interferon can effectively control human hepatitis B virus (HBV) infection, complete cure of HBV infection is quite rare. Targeting host factors involved in the viral life cycle contributes to developing innovative therapeutic strategies to improve HBV clearance. In this study, we found that the mRNA and protein levels of SIRT2, a class III histone deacetylase, were significantly upregulated in CHB patients, and that SIRT2 protein level was positively correlated with HBV viral load, HBsAg/HBeAg levels, HBcrAg, and ALT/AST levels. Functional analysis confirmed that ectopic SIRT2 overexpression markedly increased total HBV RNAs, 3.5-kb RNA and HBV core DNA in HBV-infected HepG2-Na^+^/taurocholate cotransporting polypeptide cells and primary human hepatocytes. In contrast, SIRT2 silencing inhibited HBV transcription and replication. In addition, we found a positive correlation between SIRT2 expression and HBV RNAs synthesis as well as HBV covalently closed circular DNA transcriptional activity. A mechanistic study suggested that SIRT2 enhances the activities of HBV enhancer I/HBx promoter (EnI/Xp) and enhancer II/HBc promoter (EnII/Cp) by targeting the transcription factor p53. The levels of HBV EnI/Xp and EnII/Cp-bound p53 were modulated by SIRT2. Both the mutation of p53 binding sites in EnI/Xp and EnII/Cp as well as overexpression of p53 abolished the effect of SIRT2 on HBV transcription and replication. In conclusion, our study reveals that, in terms of host factors, a SIRT2-targeted program might be a more effective therapeutic strategy for HBV infection.

## Introduction

Human hepatitis B virus (HBV) infection is a major public health problem worldwide. Despite hepatitis B vaccination, which has halted virus transmission to a large extent, approximately 250 million individuals currently live with chronic hepatitis B infection. These patients are at high risk of suffering from severe liver diseases such as liver cirrhosis and primary hepatocellular carcinoma (HCC; Martinez et al., 2020). Therefore, analyzing the HBV life cycle regulatory network is of great significance for elucidating the pathogenesis of HBV infection and reducing the occurrence of HBV-related liver diseases. As a hepatotrophic DNA virus, HBV entry into host hepatocytes is mediated by the HBV receptor Na+/taurocholate cotransporting polypeptide (NTCP; Yan et al., 2012). Subsequently, the HBV genome, termed relaxed circular DNA (rcDNA), enters the nucleus to form covalently closed circular DNA (cccDNA), which exists as a minichromosome comprised of a variety of histones and nonhistones (Allweiss and Dandri, 2017). covalently closed circular DNA serves as the template for HBV transcription and replication. The interaction between HBV and host cells is important for maintaining persistent HBV infection. Many studies have shown that histone modification, RNA polymerase II, and transcription factors contribute to regulating the transcriptional activity of cccDNA (Xia and Guo, 2020). In addition, many host factors, such as hepatocyte nuclear factor 4α (HNF4α), cAMP-response element binding protein (CREB), silent mating type information regulation 2 homolog 1 (SIRT1), and tumor protein p53 (p53) can bind to HBV genome regulatory elements and participate in the regulation of HBV transcription and replication (Tacke et al., 2005; Ren et al., 2014; Chu et al., 2016; Teng et al., 2021). Although a growing number of studies have focused on regulation of the HBV life cycle, the regulatory mechanism of HBV replication remains quite obscure.

The sirtuin family contains seven members (SIRT1–7), which are class III histone deacetylases that depend on nicotinamide adenine dinucleotide (NAD^+^; Blander and Guarente, 2004). Previous studies have demonstrated that sirtuins participate in the modulation of multiple physiological activities, including cell metabolism, the aging process, DNA repair, the cell cycle, apoptosis and the inflammatory response (North and Verdin, 2004; Chen et al., 2019; Zhang et al., 2019). In recent years, the interaction between sirtuins and viral infection has been increasingly reported. Our published studies have also uncovered the relationship between several members of the sirtuin family and regulation of the HBV life cycle. Both SIRT1 and SIRT6 promote HBV transcription and replication by enhancing the activity of the HBV core promoter, which is mediated through the transcription factors activator protein-1(AP-1) and peroxisome proliferator-activated receptor alpha (PPARα; Ren et al., 2014; Jiang et al., 2019), respectively. In contrast, SIRT3 and SIRT7 were found to restrict HBV transcription and replication through epigenetic regulation of the cccDNA minichromosome (Ren et al., 2018; Yu et al., 2021). As a member of sirtuin, SIRT2 can regulate the acetylation, phosphorylation, and cellular localization of substrates (Blander and Guarente, 2004). Such functions are directly related to the stability and activity of proteins involved in mitosis regulation, genome integrity, cellular homeostasis, aging, the oxidative stress response, inflammation, and tumorigenesis (Wang et al., 2019). Moreover, the role of SIRT2 in viral infection has also been gradually revealed. SIRT2 was recently found to be a potential target against dengue infection (Wan et al., 2021). Additionally, SIRT2 has been reported to modulate the HBV life cycle, and one inhibitor of SIRT2 has been confirmed to have an anti-HBV effect (Yu et al., 2018). Nonetheless, the mechanism underlying the regulatory role of SIRT2 on HBV transcription and replication is still poorly understood.

In the present study, we confirmed the role of SIRT2 in HBV transcription and infection. Then, expression of SIRT2 in CHB patients, coupled with the relationship between SIRT2 expression and HBV infection was also assessed. A mechanistic study found that SIRT2 enhanced the activities of HBV enhancers and promoters by inhibiting expression of the transcription factor p53, resulting in an increase in HBV cccDNA transcriptional activity and HBV RNA synthesis. Our study extends the understanding of the essential nature of SIRT2 in HBV infection and highlights that SIRT2 could serve as a potential target for CHB treatment.

## Materials and Methods

### Patients

Twenty-five patients with CHB and 25 healthy volunteers were recruited from Chongqing Traditional Chinese Medicine Hospital. The diagnosis criteria of CHB is consistent with the Guideline of Prevention and Treatment for Chronic Hepatitis B of China. The CHB patients were treatment-naïve. Subjects had autoimmune liver disease, alcohol-related liver disease, non-alcoholic steatohepatitis or coinfection with hepatitis C virus, hepatitis D virus, or human immunodeficiency virus, were excluded. The serum in the whole blood samples of the CHB patients and healthy volunteers was separated by centrifugation at 2,000 rpm for 15 min, and peripheral blood mononuclear cells (PBMCs) in the whole blood samples were isolated according to the instructions from the PBMC isolation kit (Tbdscience, Tianjin, China). Informed consent was obtained from all patients and healthy volunteers. This research study was approved by the Ethics Committee of Chongqing Medical University.

### Cell Culture, Antibodies and Reagents

Huh-7 cells were purchased from the Cell Bank of the Chinese Academy of Sciences (Shanghai, China) and maintained in Dulbecco’s modified Eagle’s medium (DMEM, Sigma) supplemented with 10% fetal bovine serum (Gibco, United States). HepAD38 and HepG2-NTCP cells were obtained from Xiamen University, HepAD38 cells were maintained in DMEM supplemented with 10% FBS and 400 μg/ml G418, and HepG2-NTCP cells were maintained in DMEM supplemented with 10% FBS, 2 μg/ml puromycin and 2 μg/ml Dox. PHHs were purchased from ScienCell and were maintained in hepatocyte medium (ScienCell, Carlsbad, CA, United States). All cells were incubated in a humidified atmosphere at 37°C and 5% CO_2_. Transfection was performed using Lipofectamine^™^ 3,000 (Invitrogen, United States) according to the manufacturer’s protocol. Rabbit anti-β-actin polyclonal antibody (100 μg/ml; sc-1, 616-R) and mouse anti-SIRT2 monoclonal antibody (200 μg/ml; sc-28, 298) were purchased from Santa Cruz Biotechnology (Dallas, TX, United States), and mouse anti-p53 monoclonal antibody (72 μg/ml; #48818) was obtained from Cell Signaling Technology (United States). The Human Sirtuin 2 (SIRT2) ELISA Kit was obtained from Abbexa Ltd. (abx383209; Cambridge, United Kingdom).

### HBV Infection

Hepatitis B virus particles from the culture supernatant of HepAD38 cells were precipitated with 5% PEG8000 overnight at 4°C, followed by centrifugation at 4,000 g for 30 min and resuspension in Opti-MEM. The HBV DNA levels in concentrated HBV particles were measured using real-time PCR. HBV particle-infected HepG2-NTCP cells and PHHs were cultured as previously described. The cells were seeded into 6-well plates and cultured with growth medium for 24 h. The cells were then maintained in PMM containing 2 μg/ml Dox for 24 h, followed by infection with HBV particles at a multiplicity of infection (MOI) of 1,000 in the presence of 4% PEG 8000. After 16 h, the cells were washed three times with PBS and maintained in growth medium with 2.5% DMSO.

### Northern Blot

Cellular RNA was extracted using TRIzol reagent. The HBV RNA levels in HBV-infected cells were detected using a DIG Northern Starter Kit (Roche, Switzerland) according to the manufacturer’s instructions. Briefly, 10 μg of total RNA were separated by electrophoresis in a 1.4% formaldehyde-agarose gel and stained with ethidium bromide to assess the levels of 28S and 18S rRNAs, which were the loading controls, followed by transfer onto a positively charged nylon membrane (GE Health care, United Kingdom). Then, the membrane was hybridized with a DIG-labeled (−) strand HBV RNA probe corresponding to nucleotides 126–1,225 of the HBV genome. After hybridization with the probe, the membrane was washed and blocked, and HBV RNA was then detected using CDP-Star and visualized with autoradiography film.

### Quantitative Real-Time PCR Analysis

Total RNA was isolated by using TRNzol Universal reagent (TIANGEN, China). Total RNA (1 μg) was reverse transcribed into cDNA using the FastKing RT Kit (with gDNase; TIANGEN, China) according to the manufacturer’s protocol. Targeted RNA levels were analyzed *via* qPCR using iTaqTM Universal SYBR^®^ Green Supermix (Bio–Rad, United States), and β-actin mRNA served as an internal control. Relative RNA levels were calculated as 2^−ΔΔ*Ct*^. All experiments were repeated three times. The primers used in the experiments are listed in Supplementary Table 1.

### HBV Core DNA Extraction and Quantitative Analysis

The cells were washed and lysed with lysis buffer (10 mM Tris–HCl pH 8.0, 1 mM EDTA, 1% NP-40, 2% sucrose). Following centrifugation, the supernatants containing cytoplasmic cell lysate were digested with DNase. The HBV core particles were precipitated using 5% PEG8000 and then incubated with proteinase K to release HBV core DNA. Then, phenol/chloroform and isopropanol were used to extract and precipitate HBV core DNA, respectively. HBV core DNA levels were measured using real-time PCR with FastStart Universal SYBR Green Master Mix (Roche, Switzerland). A series of dilutions of pCH9/HBV1.1 served as standards for absolute quantification.

### Southern Blot

The extracted HBV core DNA samples were separated on a 0.9% agarose gel and transferred to a nylon membrane (GE Health care, United Kingdom). After ultraviolet crosslinking and prehybridization, the membrane was probed with “DIG-labeled probes of full-length HBV genome.” Then, the membrane was blocked and incubated with anti-DIG antibody. The signal was visualized using autoradiography film.

### Nascent RNA Synthesis Assay

Nascent RNA synthesis assays were performed using a Click-iT^®^ Nascent RNA Capture Kit (Thermo, United States). In brief, the cells were incubated with 0.2 mM 5-ethynyl uridine (EU) for 24 h. Then, the total cellular RNA was isolated using an RNeasy Plus Mini kit (Qiagen, United States). Nascent RNA labeled with EU was biotinylated through a click reaction. The biotinylated RNA was captured by streptavidin-coated beads. Using the beads as the template, cDNA synthesis was performed using a FastKing RT Kit (with gDNase; TIANGEN, China), followed by releasing cDNA from the beads at 85°C for 5 min. Then, the nascent RNA levels were detected *via* qPCR using iTaqTM Universal SYBR^®^ Green Supermix (Bio–Rad, United States), and β-actin mRNA served as an internal control.

### Western Blot

Total cellular protein was extracted using RIPA lysis buffer with protease inhibitor. The protein concentration was determined using a BCA protein assay kit (Thermo Fisher, United States). Each sample (30 μg) was separated on SDS–polyacrylamide gels and transferred onto a polyvinylidene fluoride membrane (GE Health care, United Kingdom). The membrane was blocked in 5% skim milk and incubated with primary antibodies overnight at 4°C, followed by exposure to secondary antibodies at room temperature for 2 h. The protein signal was visualized using ECL Western blot reagents (Millipore, MA, United States).

### HBV cccDNA Extraction and Analysis

HBV cccDNA was extracted using the Hirt method. Briefly, cells were collected into a 1.5 ml tube and lysed in 0.5 ml SDS lysis buffer at room temperature for 30 min. Then, 0.125 ml of 2.5 mol/l KCl was added, and the tube was gently inverted ten times, followed by incubation at 4°C overnight. The sample was centrifuged at 14,000 g for 20 min. The Hirt DNA in the supernatant was purified using phenol and phenol/chloroform. For cccDNA detection, the Hirt DNA was heated at 85°C for 5 min and digested by T5 exonuclease (New England Biolabs, United States) at 37°C for 1 h, followed by heating at 100°C for 20 min. The HBV cccDNA levels in the treated Hirt DNA were assessed by Taqman-probe specific real-time PCR.

### Luciferase Reporter Assay

Cells were transfected with HBV pGL3-EnI/Xp, pGL3-EnII/Cp, pGL3-Sp1, and pGL3-Sp2 combined with lentivirus expressing SIRT2 or shSIRT2. Thirty-six hours after transfection, luciferase activity was analyzed using a dual-luciferase reporter assay kit according to the manufacturer’s instructions (Promega, United States). HBV promoter activity was standardized to RL-TK luciferase activity.

### Chromatin Immunoprecipitation

Chromatin immunoprecipitation (ChIP) assays were performed according to the manufacturer’s protocol (Merck Millipore, Germany) with minor modifications. Briefly, cells were resuspended in cell lysis buffer. Following centrifugation, the nuclei were precipitated and were fixed in 20 mM Tris at pH 8 with 3 mM MgCl_2_, and 20 mM KCl buffer containing 1% formaldehyde and protease inhibitor. Then, the cross-linked nuclei were pelleted and lysed in nuclear lysis buffer. The chromatin solution was sonicated to generate approximately 200–1,000 bp DNA fragments. After centrifugation, a portion of the supernatant was diluted 1:10 in dilution buffer, and another 5 μl supernatant was reserved as input. The diluted chromatin was then subjected to immunoprecipitation using the indicated antibodies. After reverse cross-linking, the immunoprecipitated chromatin was purified using spin columns and analyzed by real-time PCR. *glyceraldehyde-3-phosphate dehydrogenase (GAPDH)* and *myosin heavy chain 6 (MYH6)* were used as control genes. The specific primers are listed in the Supplementary Table 1.

### Statistical Analysis

The comparison of mean between two groups was conducted by using unpaired t test. Pearson’s test was used to evaluate the correlation coefficiency (*r*) of the serum SIRT2 and clinical biomarkers (HBV viral load, HBsAg, HBeAg, ALT and AST levels). The results from three independent experiments in cell models are expressed as the mean ± SD. Statistics were performed using the nonparametric Mann–Whitney U test. All statistical analyses were performed using SPSS 19.0 software. A value of *p* < 0.05 was considered significant (^*^p < 0.05; ^**^p < 0.01; n.s., not significant).

## Results

### SIRT2 Is Positively Correlated With Chronic HBV Infection

**Figure 1:**
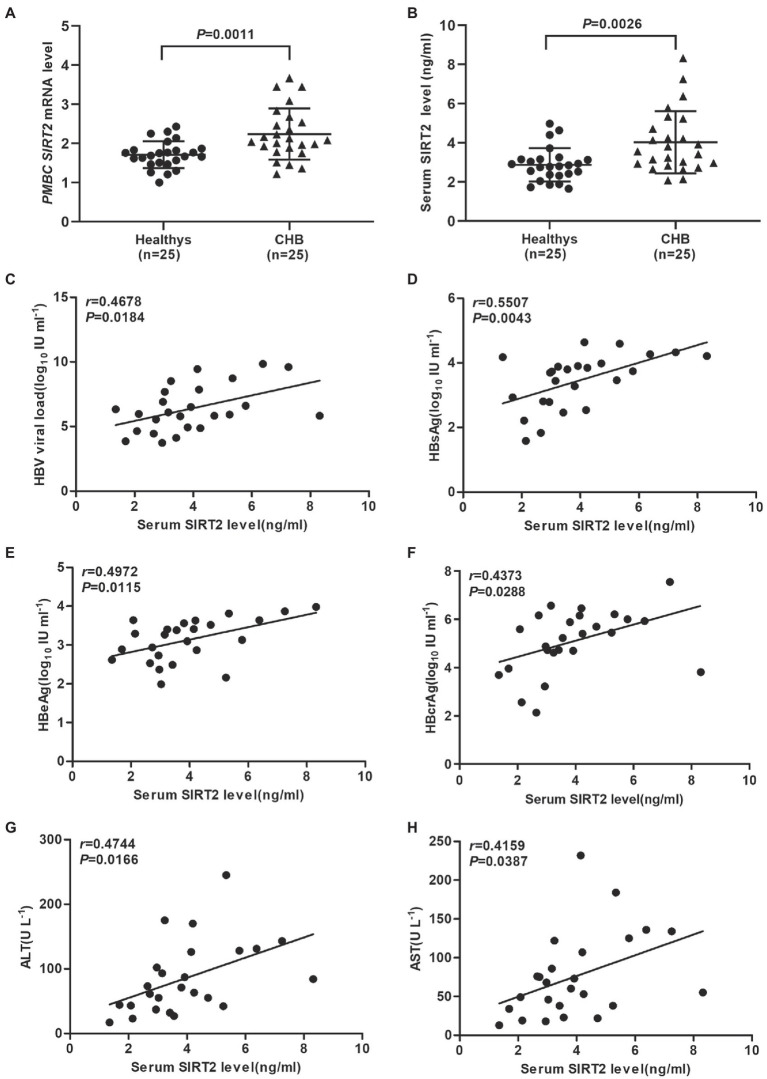
SIRT2 is correlated with chronic HBV infection. **(A,B)** The mRNA and protein levels of SIRT2 from PBMC or serum in CHB patients and healthy individuals were analyzed by real-time PCR and ELISA, β-actin was used as an internal control in real-time PCR. **(C–E)** Correlation between serum SIRT2 level and HBV viral load **(C)**, HBsAg **(D)**, HBeAg levels **(E)** and HBcrAg **(F)**. HBcrAg level in the serum of CHB patients was detected by fully automated lumipulse chemiluminescence enzyme immunoassay system (Fujirebio Inc., Tokyo, Japan). The HBV viral load, HBsAg and HBeAg levels were log_10_ transformed. The correlation co-efficiency (*r*) and two-tailed *p* values were calculated *via* Pearson correlation. **(G,H)** Analysis of correlation between serum SIRT2 level and transaminase ALT, and AST levels using Pearson’s test.

Evidence has shown that HBV replication might contribute to SIRT2 expression (Cheng et al., 2018). To further explore the clinical significance of SIRT2 in HBV infection, the mRNA levels of SIRT2 in PBMCs from 25 CHB patients and 25 healthy volunteers were examined using real-time PCR. The clinical characteristics of the patients and healthy volunteers are shown in Table 1. Our results suggested that SIRT2 mRNA levels in PBMCs from CHB patients were significantly higher than that in PBMCs from healthy individuals (*p* = 0.0011, Figure 1A). Consistently, protein levels of SIRT2 were elevated in the serum of CHB patients compared to healthy people, as demonstrated by quantitative ELISA (*p* = 0.0026, Figure 1B). Correlation analysis revealed that serum SIRT2 levels were positively correlated with HBV viral load (Pearson correlation coefficient, *r* = 0.4678, *p* = 0.0184, Figure 1C), HBsAg levels (Pearson correlation coefficient, *r* = 0.5507, *p* = 0.0043, Figure 1D), HBeAg levels (Pearson correlation coefficient, *r* = 0.4972, *p* = 0.0115, Figure 1E) and HBcrAg levels (Pearson correlation coefficient, *r* = 0.4373, *p* = 0.0288, Figure 1F). Further investigation found that transaminase ALT and AST levels were positively correlated with serum SIRT2 levels in CHB (Pearson correlation coefficient, *r* = 0.4744, *p* = 0.0166 and Pearson correlation coefficient, *r* = 0.4159, *p* = 0.0387, Figures 1G,H). These findings suggested that SIRT2 might play a role in the HBV infection process.

**Table 1:** Clinical characteristic of heathy volunteers and patients with CHB.

Characteristics	Healthy (25)	CHB (25)	*p (<0.05)
Age (years)	30.84 ± 9.21	29.48 ± 7.66	0.5730 (ns)
Gender (M/F)	10/15	14/11	0.396 (ns)
ALT (U/L)	17.88 ± 12.74	85.08 ± 56.17	0.0001***
AST (U/L)	17.96 ± 5.97	75 ± 54.95	0.0001***
ALB (g/L)	43.26 ± 6.94	42.01 ± 4.34	0.0297*
GLB (g/L)	28.31 ± 2.93	31.06 ± 3.54	0.0009***
TBIL (μmol/L)	14.05 ± 6.93	20.75 ± 21.88	0.1903 (ns)
AFP (ng/mL)	2.52 ± 2.62	4.04 ± 8.11	0.8513 (ns)

* *p < 0.05*,

*** *p < 0.001*,

*ns, not significant*.

*All values are expressed as the mean ± standard deviation*.

*F, female; M, male; ALT, alanine aminotransferase; AST, aspartate aminotransferase; ALB, albumin; GLB, globulin; TBIL, total bilirubin; AFP, alpha fetoprotein*.

### SIRT2 Overexpression Facilitates HBV Transcription and Replication

**Figure 2:**
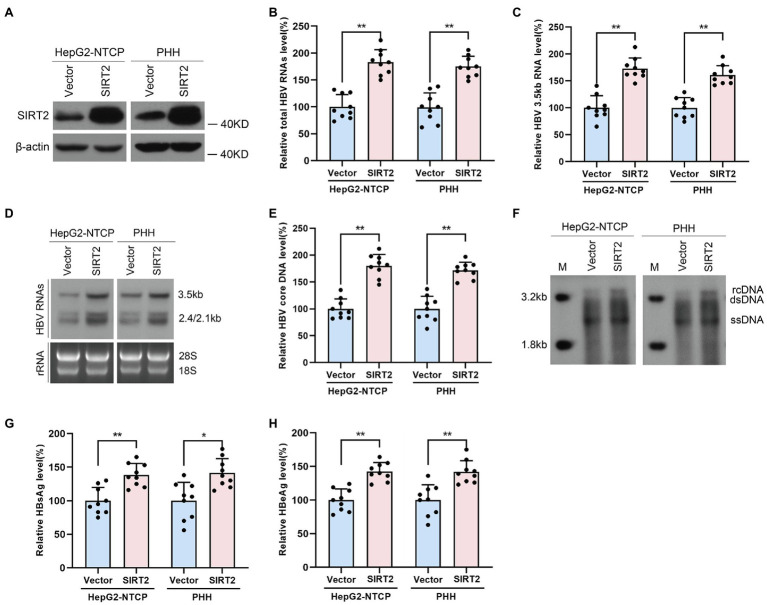
Ectopic expression of SIRT2 promotes HBV transcription and replication. HepG2-NTCP cells or PHH were infected with 1 × 10^3^ genome equivalents/cells of HBV for 16 h and then transduced with lentivirus expressing SIRT2 isoform 1. Cells were harvested to test the overexpression efficiency and examine the viral markers after 5 days. **(A)** The overexpression efficiency of SIRT2 was determined by Western blot. **(B–D)** SIRT2 overexpression elevated total HBV RNAs level and HBV 3.5-kb RNA level, as demonstrated by real-time PCR **(B,C)** and Northern blot **(D)**. Each panel was loaded with an equal amount of total RNA and the HBV RNAs were hybridized with DIG-labed single-stranded HBV RNA probe with a length of 1,000 bp. Ribosomal RNAs (28 s and 18 s) served as loading controls. **(E,F)** SIRT2 overexpression enhanced HBV core DNA level according to real-time PCR **(E)** and Southern blot **(F)**. **(G,H)** HBsAg and HBeAg levels were measured by ELISA. Representative data are from at least three independent experiments. Data was shown as mean ± SD. ^*^p < 0.05, ^**^p < 0.01.

To confirm the potential role of SIRT2 in HBV infection, HepG2-NTCP cells or PHHs were transduced with lentivirus expressing SIRT2 isoform 1 after infection with HBV particles. Western blot was performed to verify the overexpression efficiency of SIRT2 (Figure 2A). Real-time PCR analysis showed that ectopic expression of SIRT2 led to remarkably increased total HBV RNAs and HBV 3.5-kb RNA levels in both cell lines (Figures 2B,C). Northern blot analysis indicated that HBV 3.5-kb, 2.4-kb, and 2.1-kb RNA levels were increased in SIRT2-overexpressing cells (Figure 2D). Consistently, high expression of SIRT2 also increased the levels of HBV core DNA, as evidenced by real-time PCR and Southern blot (Figures 2E,F). Moreover, ELISA revealed that SIRT2 overexpression promoted HBsAg and HBeAg secretion levels (Figures 2G,H). In addition, a timeline for the role of SIRT2 in regulating HBV transcription and replication was furtherly studied. The results showed that on the 5th and 7th days after SIRT2 overexpression, levels of HBV RNAs, HBV core DNA, HBsAg and HBeAg in HepG2-NTCP cells were significantly increased. However, on the 3rd day after SIRT2 overexpression, the indicators mentioned above that associates with HBV transcription and replication were not evidently changed (Supplementary Figure S1). Collectively, these results indicated that SIRT2 overexpression significantly promotes HBV transcription and replication.

### SIRT2 Silencing Suppresses HBV Transcription and Replication

**Figure 3:**
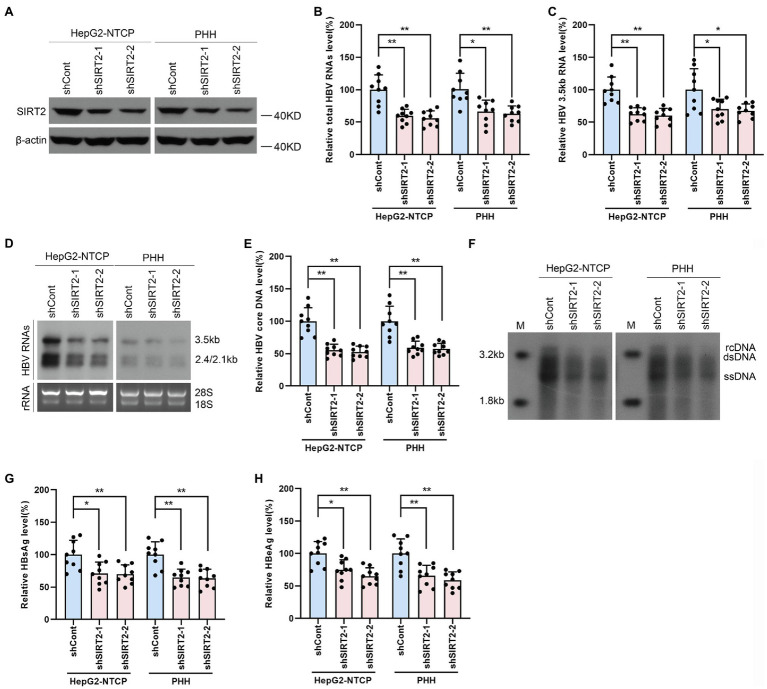
Gene silencing of SIRT2 inhibits HBV transcription and replication. HepG2-NTCP cells or PHH were infected with 1 × 10^3^ genome equivalents/cells of HBV for 16 h and then transduced with lentivirus expressing indicated shRNA (shSIRT2-1 and shSIRT2-2) and scramble control (shCont). Cells were harvested to test the knockdown efficiency and examine the viral markers after 5 days. **(A)** The efficiency of SIRT2 silencing was examined *via* Western blot. **(B–D)** SIRT2 suppression decreased total HBV RNAs level and HBV 3.5-kb RNA level based on real-time PCR **(B,C)** and Northern blot **(D)**. Each panel was loaded with an equal amount of total RNA and the HBV RNAs were probed with DIG-labeled single-stranded HBV RNA probe with a length of 1,000 bp. Ribosomal RNAs (28 s and 18 s) were used as loading controls. **(E,F)** SIRT2 knockdown suppressed HBV core DNA level as determined by real-time PCR **(E)** and Southern blot **(F)**. **(G,H)** HBsAg and HBeAg levels were detected by ELISA. Representative data are from at least three independent experiments. Data are shown as mean ± SD. ^*^p < 0.05, ^**^p < 0.01.

To further investigate whether endogenous SIRT2 exerts a functional effect on HBV transcription and replication, we inhibited SIRT2 expression using shRNA targeting SIRT2 (shSIRT2-1 and shSIRT2-2). The silencing efficiency was assessed by Western blot (Figure 3A). SIRT2 knockdown decreased total HBV RNAs and HBV 3.5-kb RNA levels in both HepG2-NTCP cells and PHHs (Figures 3B,C). Northern blot confirmed that HBV 3.5-kb, 2.4-kb, and 2.1-kb RNA levels were decreased in response to SIRT2 depletion (Figure 3D). In addition, real-time PCR and Southern blot also indicated that SIRT2 suppression significantly reduced HBV core DNA levels (Figures 3E,F). Not surprisingly, ELISA indicated that SIRT2 depletion also decreased HBsAg and HBeAg levels in the supernatant (Figures 3G,H). These results suggested that silencing SIRT2 inhibits HBV transcription and replication.

### Overexpression of SIRT2 Enhances HBV cccDNA Transcriptional Activity

**Figure 4:**
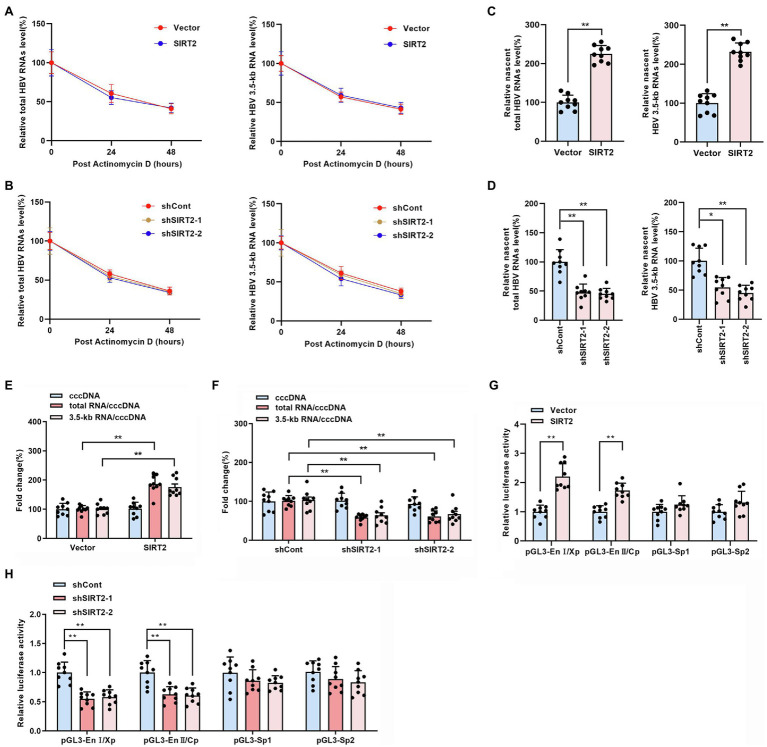
SIRT2 regulates HBV cccDNA transcription activity. **(A,B)** HepG2-NTCP cells were infected with HBV for 16 h and then transduced with indicated lentivirus for 4 days and treated with Actinomycin D (5 μg/ml). Cells were harvested at indicated time. Total HBV RNAs **(A)** and HBV 3.5-kb RNA **(B)** were examined by real-time PCR, and the amount of RNA at time zero was set as 100%. **(C,D)** HBV-infected HepG2-NTCP cells were incubated with 0.2 mM 5-ethynyl uridine (EU) for 24 h. The newly synthesized EU-labeled RNA was purified from the total RNA, and EU-labeled HBV RNAs were quantified by real-time PCR. **(E,F)** HBV infected-HepG2-NTCP cells were transduced with indicated lentivirus. 5 days later, HBV cccDNA was extracted with Hirt lysis buffer and analyzed *via* Taqman-probe specific real-time PCR. The ratios of total RNA/cccDNA and 3.5-kb RNA/cccDNA indicated the transcriptional activity of cccDNA. **(G,H)** The effect of SIRT2 overexpression or down-regulation on HBV EnI/Xp, EnII/Cp, Sp1 and Sp2 were determined by dual-luciferase reporter assay. The transfection efficiency was normalized under co-transfection with plasmid RL-TK. Representative data are from at least three independent experiments. Data are shown as mean ± SD. ^*^p < 0.05, ^**^p < 0.01.

The above findings indicated that SIRT2 facilitates HBV transcription and replication. To further explore how HBV RNA levels were regulated by SIRT2, RNA decay assays and nascent RNA capture assays were performed. HepG2-NTCP cells were treated with actinomycin D, a chemical molecule that blocks RNA synthesis by inhibiting DNA-dependent RNA polymerase. According to the results, neither overexpression (Figure 4A) or silencing (Figure 4B) of SIRT2 affected the half-life of total HBV RNAs or HBV 3.5-kb RNA, indicating that the degradation rate of HBV RNA is not affected by SIRT2. In addition, the nascent RNA synthesis assay data demonstrated that nascent total HBV RNAs and HBV 3.5-kb RNA were increased in response to SIRT2 overexpression (Figure 4C) and decreased in response to SIRT2 knockdown (Figure 4D). The above data demonstrate that the alteration of total HBV RNAs levels is caused by the effect of SIRT2 on HBV RNA synthesis rather than HBV RNA stability. In view of HBV cccDNA as the template of HBV RNA synthesis, we further examined the effect of SIRT2 on HBV cccDNA levels and transcriptional activity. The results revealed that cccDNA transcription activity, not cccDNA levels, was affected by SIRT2 (Supplementary Figures S2A,B; Figures 4E,F). However, the cccDNA transcription activity was increased by SIRT2 overexpression and decreased by SIRT2 knockdown (Figures 4E,F). Since SIRT2 is a well-known deacetylase (Blander and Guarente, 2004), coupled with the critical role of epigenetic regulation on cccDNA transcription (Hong et al., 2017), we speculated that SIRT2 might affect the acetylation levels of cccDNA-bound H3/H4. However, we did not observe any changes in the levels of acetylated cccDNA-bound H3/H4 using a ChIP assay. The host genes *GAPDH* and *MYH6* served as controls (Supplementary Figure S3). Since cccDNA transcription activity is regulated by HBV promoters (Cp, Xp, Sp1, and Sp2) and HBV enhancer I/II, we performed a dual luciferase reporter assay to examine the effect of SIRT2 on these cis-acting elements. Interestingly, the results suggested that SIRT2 overexpression enhanced the activities of HBV EnI/Xp and EnII/Cp, while the activities of Sp1 and Sp2 were slightly affected (Figure 4G). Conversely, downregulation of SIRT2 induced the opposite effect (Figure 4H). Taken together, these results indicated that SIRT2 might regulate HBV transcription by upregulating the activity of HBV promoters and enhancers.

### SIRT2 Downregulates p53 Levels by Inhibiting the Promoter Activity of p53

**Figure 5:**
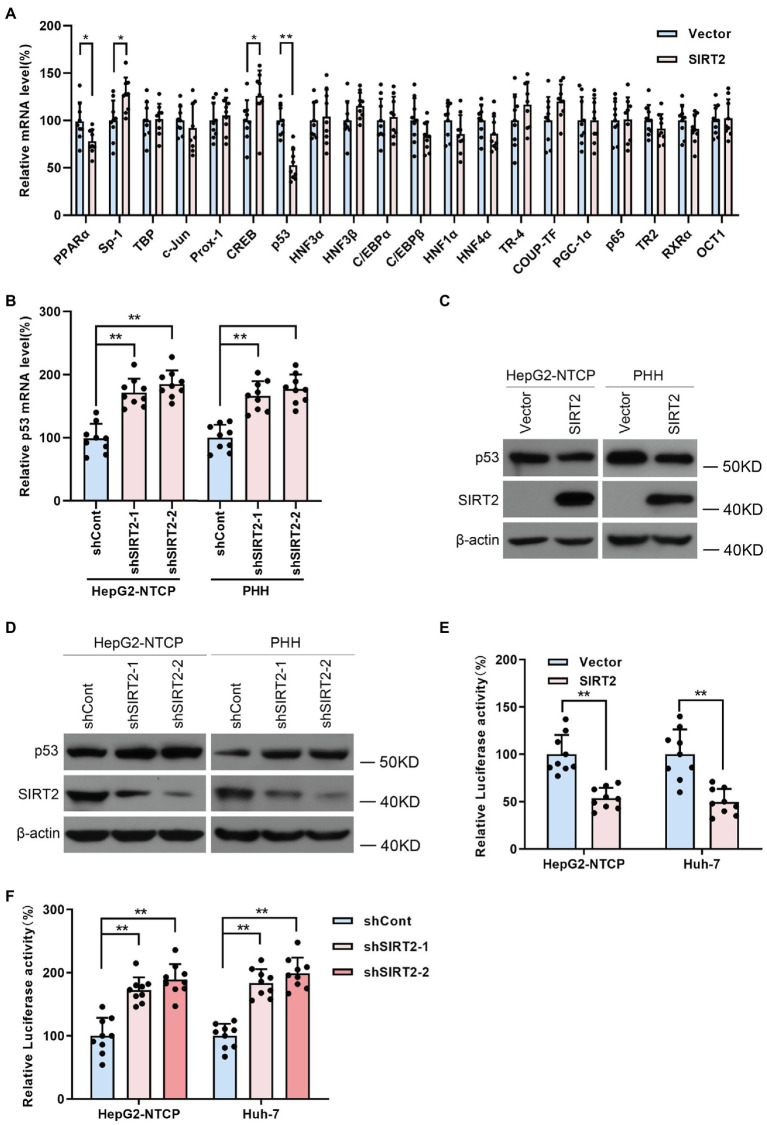
SIRT2 regulates p53 level by targeting the p53 promoter. **(A)** Real-time PCR analysis of gene expression of various transcription factors associated with HBV transcription. **(B)** HepG2-NTCP and PHH cells were transduced with lentivirus expressing shSIRT2-1/shSIRT2-2 and shCont. The effect of SIRT2 knockdown on p53 mRNA level was analyzed by real-time PCR. **(C,D)** The effect of SIRT2 overexpression or SIRT2 depletion on p53 protein level in HepG2-NTCP and PHH cells was examined by Western blot. β-actin served as the loading control. **(E,F)** HepG2-NTCP and Huh-7 cells were transfected with pGL3-p53 promoter and transfected with plasmids expressing SIRT2 or transduced with indicated lentivirus expressing shSIRT2. The luciferase activity was measured at 36 h post transfection using dual-luciferase reporter assay. Representative data are from at least three independent experiments. Data are shown as mean ± SD. ^*^p < 0.05, ^**^p < 0.01.

Considering that host transcription factors are closely related to HBV promoters and enhancers, we proposed that SIRT2 might play a role as a transcriptional promoting factor; thus, we screened a panel of transcription factors, such as nuclear factor 1 (NF-1), Sp1 transcription factor (Sp-1), TATA-box binding protein (TBP), AP-1, prospero homeobox 1 (Prox-1), CREB, p53, and hepatocyte nuclear factor 3α (HNF3α), using real-time PCR (Figure 5A). The data revealed that NF-1, Sp-1, CREB, and p53 levels were affected by SIRT2 overexpression. Given that p53 was the most downregulated in SIRT2-overexpressing cells, we further confirmed whether p53 mediated the regulation of HBV transcription by SIRT2. Consistently, we observed that SIRT2 knockdown increased p53 mRNA levels in HepG2-NTCP and PHH cells (Figure 5B). Western blot also showed that p53 protein levels were decreased in SIRT2-overexpressing cells and increased in SIRT2-silenced cells (Figures 5C,D). In addition, we established a pGL3-p53 promoter luciferase reporter plasmid. The dual-luciferase assay revealed that ectopic expression of SIRT2 inhibited the activity of the p53 promoter. Conversely, SIRT2 depletion enhanced p53 promoter activity (Figures 5E,F). In general, these data suggested that SIRT2 might regulate p53 expression by suppressing p53 transcription.

### SIRT2 Facilitates HBV Transcription and Replication by Repressing the Binding of p53 to HBV EnI/Xp and EnII/Cp

**Figure 6:**
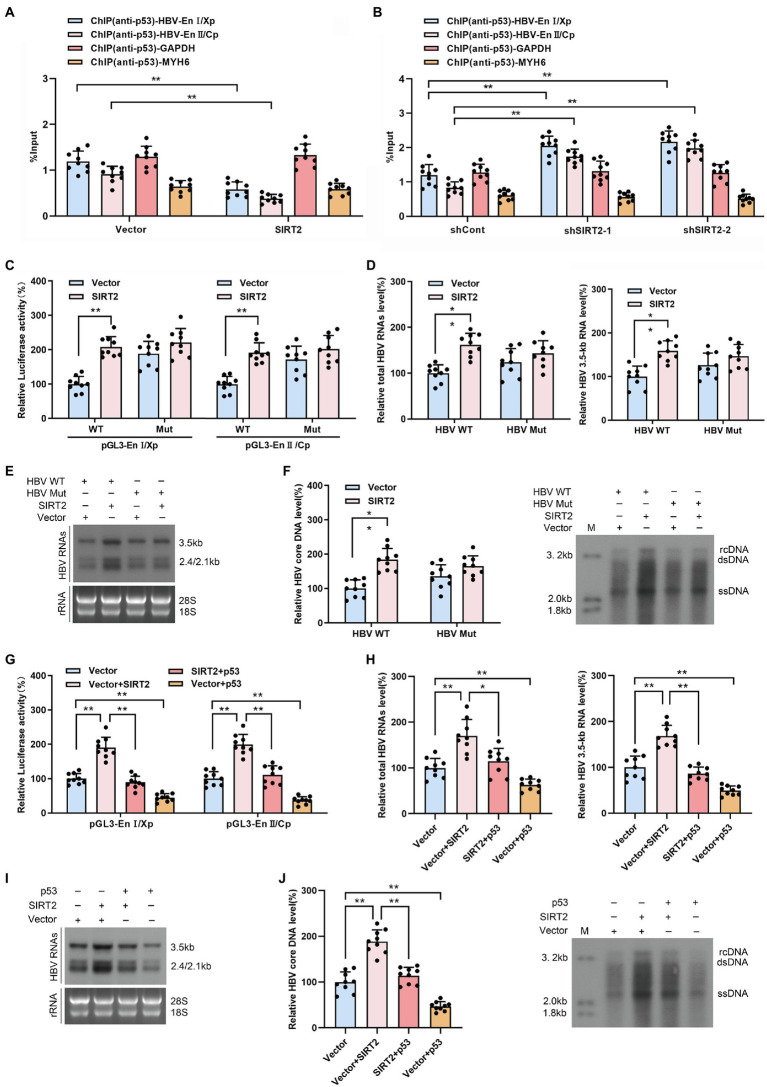
SIRT2 facilitates HBV transcription and replication *via* repressing the binding of p53 on HBV EnI/Xp and EnII/Cp. **(A,B)** Effect of SIRT2 overexpression **(A)** and down-regulation **(B)** on the recruitment of p53 to HBV EnI/Xp and EnII/Cp was detected by ChIP assay. Cross-linked chromatin from HBV-infected HepG2-NTCP cells was immunoprecipitated with anti-p53 antibody followed by real-time PCR with specific HBV EnI/Xp and EnII/Cp primers. The promoter of *GAPDH* and *MYH6* were used as internal controls. The ChIP results are expressed as % of input. **(C)** HepG2-NTCP cells were transfected with indicated plasmids. Dual-luciferase assay was performed to examine the activities of HBV EnI/Xp and EnII/Cp. pGL3-EnI/Xp Mut and pGL3-EnII/Cp Mut denoted the p53 binding sites in HBV EnI/Xp and EnII/Cp were mutated. **(D–F)** HepG2-NTCP cells were infected with HBV WT or HBV Mut (binding sites of p53 to EnI/Xp and EnII/Cp were mutated) and transduced with lentivirus expressing SIRT2. Total HBV RNAs and HBV 3.5-kb RNA levels were analyzed by real-time PCR **(D)** and Northern blot **(E)**, HBV core DNA level in HepG2-NTCP cells was analyzed by real-time PCR and Southern blot **(F)**. **(G)** HepG2-NTCP cells were transfected with indicated plasmids. Dual-luciferase assay was performed to examine the activities of HBV EnI/Xp and EnII/Cp. **(H–J)** HBV-infected HepG2-NTCP cells were transfected with plasmids containing SIRT2 or p53. Total HBV RNA and HBV 3.5-kb RNA levels were detected by real-time PCR **(H)** and Northern blot **(I)**. HBV core DNA level was measured by real-time PCR and Southern blot **(J)**. Representative data are from at least three independent experiments. Data are shown as mean ± SD. ^*^p < 0.05, ^**^p < 0.01.

HBV enhancers directly improve the efficiency of HBV promoters, and p53 can suppress HBV transcription by binding to HBV enhancer I and enhancer II (Chu et al., 2016). Therefore, we examined the effect of SIRT2 on the binding of p53 to HBV EnI/Xp and EnII/Cp in cccDNA using a ChIP assay. The data revealed that levels of HBV EnI/Xp and EnII/Cp-bound p53 were notably reduced in SIRT2-overexpressing HepG2-NTCP cells (Figure 6A) but elevated in response to SIRT2 silencing (Figure 6B). Furthermore, we mutated the p53-EnI/Xp and EnII/Cp binding sites, which have been reported in HBV EnI/Xp and EnII/Cp luciferase reporter plasmids (Chu et al., 2016; Supplementary Figures S4A,B). we found that the mutation of p53 binding sites in HBV genome could decrease the mostly binding level of p53 to EnI/Xp and EnII/Cp, and allow a moderate increase of HBV RNAs (Supplementary Figures S4C,D). Importantly, the enhancement of HBV EnI/Xp and EnII/Cp activities induced by SIRT2 overexpression was mostly abolished when the binding sites of p53 in HBV EnI/Xp and EnII/Cp were mutated (Figure 6C). In addition, we constructed a site-specific mutant virus in which the p53 binding sites were mutated. Consistently, the mutation of p53 binding sites in the HBV genome blocked the promotion of SIRT2 on HBV 2.4-kb, 2.1-kb and 3.5-kb RNA levels as well as core DNA levels (Figures 6D–F). Moreover, we found that the enhancement of SIRT2 overexpression on the activities of HBV EnI/Xp and EnII/Cp, HBV 2.4-kb, 2.1-kb and 3.5-kb RNA levels as well as core DNA levels were abolished by the introduction of p53 (Supplementary Figure S5; Figures 6G–J). Furthermore, we detected whether SIRT2 could regulate HBV transcription and replication in p53 knockout HepG2-NTCP cells. The results showed that SIRT2 overexpression could not significantly increase HBV RNAs and HBV core DNA levels in the absence of p53 (Supplementary Figure S6), implying that p53 is critical for SIRT2-mediated modulation of HBV transcription. Altogether, these data suggested that SIRT2 might positively regulate HBV transcription and replication by regulating the transcription factor p53.

## Discussion

The virus-cellular host factor interaction is a continuous process throughout the different stages of the viral life cycle (Ligat et al., 2021). Many viruses can create an intracellular environment favorable for infection by enhancing or disrupting the expression of host genes (Duggal and Emerman, 2012; Nakagawa and Makino, 2021). The interplay between virus and host factors determines viral pathogenicity and the outcome of infection (Wißing et al., 2021). Accordingly, clarifying virus–host cell interactions is critical for understanding the mechanisms of pathogenesis and developing novel antiviral therapies (Todt et al., 2018). Consistently, studies have shown that HBV–host interactions are involved in the entire HBV life cycle and that a complete HBV infection process depends on host factors (Ligat et al., 2021). For example, NTCP mediates virus entry (Herrscher et al., 2020), the DNA repair machinery functions in HBV cccDNA formation (Wei and Ploss, 2020, 2021), cellular transcription factors contribution to viral transcription (Quasdorff and Protzer, 2010; Oropeza et al., 2020), and numerous cellular factors involved in virion assembly and release (Hu and Liu, 2017; Zeyen and Prange, 2018). Hence, targeting host factors that contribute to HBV replication is a promising approach for improving the cure rate. Our previous studies have identified several host factors involved in HBV infection, such as SIRT1, SIRT6, cyclin D2, UBE2L3, NQO1, SIRT3, and SIRT7 (Ren et al., 2014, 2018; Song et al., 2014, p. 2; Jiang et al., 2019; Zhou et al., 2019; Cheng et al., 2021; Yu et al., 2021). In addition, AGK2, a SIRT2 inhibitor, was found to exert an anti-HBV effect, implying that SIRT2 might facilitate for HBV infection (Yu et al., 2018, p. 2). Consistent with this hypothesis, a study by Piracha et al. (2018) revealed that SIRT2 isoform 1 enhanced HBV transcription and replication. Moreover, they further demonstrated that SIRT2 overexpression and HBV replication activated AKT/GSK-3β/β-catenin signaling. However, there was no evidence that AKT/GSK-3β/β-catenin signaling mediated the role of SIRT2 in HBV replication. Therefore, the mechanism by which SIRT2 regulates the HBV life cycle needs to be confirmed.

In the present study, we demonstrated that compared to healthy volunteers, patients with CHB exhibited significantly higher SIRT2 mRNA levels in PBMCs. PBMCs were widely used to analyze gene expression signatures that may be related to disease predictions (Bushel et al., 2007; Hofmann et al., 2010; Gliddon et al., 2018). Some genes, which were aberrant expression in PBMCs of CHB patients were also proved to involve in the regulation of HBV life cycle, such as Ubiquitin Conjugating Enzyme E2 L3 (UBE2L3), Exportin 4 (XPO4), matrix metalloproteinase 9 (MMP-9), interferon gamma-inducible protein 16 (IFI16) and LncHOTAIR(Zhang et al., 2014; Chen et al., 2017; Zhou et al., 2019; Ren et al., 2020; Lu et al., 2021). Therefore, the result of SIRT2 aberrant expression in PBMCs of CHB patients implied SIRT2 might be associated with HBV replication. Moreover, we found the SIRT2 protein was also high expression in the serum of CHB patients and positively associated with HBV viral load, HBsAg, HBeAg, HBcrAg levels, and ALT and AST levels. These results suggest that SIRT2 might be a favorable factor for HBV replication. The function of SIRT2 in the HBV life cycle was further examined using HepG2-NTCP cells and PHHs, two widely utilized HBV infection models. Although SIRT2 was reported to be involved in regulating the proliferation of HCC cells (Chen et al., 2013), we observed that SIRT2 did not significantly affect the cell viability of HepG2-NTCP cells and PHHs under our experimental conditions (Supplementary Figure S7). As we expected, HBV RNA, HBV core DNA, and viral protein expression was significantly upregulated in response to SIRT2 overexpression, whereas SIRT2 inhibition reduced HBV transcription and replication. In addition, we confirmed that HBV RNA synthesis and cccDNA transcription activity were increased in SIRT2-overexpressing cells. cccDNA is subject to epigenetic regulation involving H3/H4 acetylation or methylation (Xia and Guo, 2020). The transcriptional activity of cccDNA is regulated by a series of histone acetyltransferases, deacetylases and methyltransferases (Hong et al., 2017). SIRT2 belongs to the class III histone deacetylase (Chen et al., 2020). Piracha et al. (2020) showed SIRT2 isoform1 and isoform 5 mainly located in cytoplasm and nucleus, respectively. And cccDNA was epigenetically regulated to a greater extent in SIRT2 isoform 5 overexpressing cells than in SIRT2 isoform1 overexpressing cells. Although SIRT2 has been established as a cytoplasmic-localized protein (Wilking-Busch et al., 2018), SIRT2 was reported to translocate into the nucleus and deacetylate histone H3K18 after microbial infection (Bhaskar et al., 2020). These findings indicated SIRT2 promoted HBV transcription may through epigenetic regulation of cccDNA. However, in this study, we did not observe redistribution of SIRT2 to the nucleus (Supplementary Figure S8), and the acetylation state of cccDNA-binding H3/H4 was not influenced by SIRT2 overexpression, implying that the regulatory effect of SIRT2 on HBV transcription may not directly toward the epigenetic modification of cccDNA minichromosome. In addition, we have reported that AGK2, the inhibitor of SIRT2 activity, could decrease HBV transcription and replication, suggesting that the role of SIRT2 in regulating HBV lifecycle may depend on its deacetylase activity. However, more data should to be provided to confirm the speculation. It is widely known that the transcription of HBV is modulated by four promoters and two enhancers (Tsukuda and Watashi, 2020). Our data confirmed that the activities of the HBV EnI/Xp and EnII/Cp were increased in SIRT2-overexpressing cells but were decreased in SIRT2-depleted cells. Hence, we speculated that the regulation of SIRT2 on HBV transcription and replication might be dependent on HBV enhancers and promoters.

To clarify how SIRT2 augments HBV enhancers and promoters’ activities, a panel of transcription factors related to HBV enhancers and promoters were screened using real-time PCR. The results showed that alteration of p53 mRNA levels were most notable after SIRT2 overexpression. In addition, we further confirmed the regulatory effect of SIRT2 on p53 protein levels and p53 promoter activity. However, we also found that SIRT2 did not influence the H3/H4 acetylation level of p53 promoter (Supplementary Figure S9), suggesting the presence of some unidentified intermediates in the regulation of SIRT2 on p53 expression. A great deal of evidence has shown that p53 is a transcription factor serving a dual function of transactivation and transrepression (Men et al., 2021). Its primary biological function appears to be involved in regulating DNA damage, oxidative stress, cell differentiation, apoptosis, cell cycle arrest and metabolism alterations (Hernández Borrero and El-Deiry, 2021; Men et al., 2021). P53 acting as a transcriptional suppressor has been demonstrated in several studies, indicating that p53 could bind to HBV enhancers and inhibit the activities of HBV enhancers and promoters (Ori et al., 1998; Chu et al., 2016). Consistently, we found the levels of HBV EnI/Xp and EnII/Cp-bound p53 were weakened in response to SIRT2 overexpression. Our data showed that the regulation of HBV transcription and replication by SIRT2 was attenuated but not completely abolished whether in p53 binding sites mutant HBV-infected HepG2-NTCP cells or in p53-deleted HepG2-NTCP cells. Additionally, overexpression of p53 attenuated the regulatory effect of SIRT2 on HBV life cycle. These findings suggested that p53 might play an important role in the regulation of SIRT2 on HBV transcription. However, other potential factors involving the regulation of HBV transcription and replication by SIRT2 could not be excluded.

Since host factors play a key role in HBV life cycle, targeting the host factors is a feasible approach to develop new anti-HBV strategies. Based on the important role of SIRT2 in HBV transcription and replication, inhibiting SIRT2 expression or activity contributes to disturb the HBV replication process. At present, various SIRT2-selective inhibitors have been found. AGK2, the IC50 value for SIRT2 is 3.5 μM (Outeiro et al., 2007), has been proved to be able to significantly decrease HBV RNAs and HBV DNA levels (Yu et al., 2018). Thiomyristoyl (TM), another SIRT2-specific inhibitor, the IC50 value for SIRT2 is 28 nM (Jing et al., 2016). Compared to AGK2, TM exhibited a 125-fold lower IC50 value, implying that TM has a more potent ability to inhibit HBV transcription and replication. Interestingly, Tenovin-3, a SIRT2 inhibitor, could simultaneously activate p53 expression (Lain et al., 2008). These findings suggest that it is worthwhile to furtherly study the potential antiviral effect of SIRT2 inhibitors, and the combination of available anti-HBV drugs and SIRT2 inhibitors may contribute to produce more effective virological response.

The current study reconfirmed the facilitating function of SIRT2 in HBV transcription and replication using HBV-infected cell models. The results from clinical samples also revealed a positive association between SIRT2 expression and HBV infection. We further identified that SIRT2 enhances the activities of HBV EnI/Xp and EnII/Cp by downregulating the expression of p53, boosting the transcriptional activity of HBV cccDNA. This study reinforces the significance of the interplay between virus and host factors, suggesting that intervention in this interaction might represent a promising antiviral strategy.

## Data Availability Statement

The original contributions presented in the study are included in the article/supplementary material, further inquiries can be directed to the corresponding author.

## Ethics Statement

Written informed consent was obtained from the individual(s) for the publication of any potentially identifiable images or data included in this article.

## Author Contributions

J-HR designed the study, D-QW, Q-YD, and M-LD performed experiments and analyzed the data. S-TC, FR, FL, and JC provided help in performing the research. N-NT, MT, and YZ collected the clinical specimens and analyzed the patient data. J-HR, D-QW, and Y-JZ wrote the manuscript. All authors contributed to the article and approved the submitted version.

## Funding

This research was funded by National Natural Science Foundation of China (81802015), Scientific and Technological Research Program of Chongqing Municipal Education Commission (KJQN202100429), College Young Teachers Fund of the Fok Ying Tung Education Foundation (171100), Natural Science Foundation Project of Chongqing (cstc2019jscx-dxwtBX0020), and Chongqing Talents Project (cstc2021ycjh-bgzxm0121).

## Conflict of Interest

The authors declare that the research was conducted in the absence of any commercial or financial relationships that could be construed as a potential conflict of interest.

## Publisher’s Note

All claims expressed in this article are solely those of the authors and do not necessarily represent those of their affiliated organizations, or those of the publisher, the editors and the reviewers. Any product that may be evaluated in this article, or claim that may be made by its manufacturer, is not guaranteed or endorsed by the publisher.

## Abbreviations

HBV: Hepatitis B virus
HCC: Hepatocellular Carcinoma
NTCP: Na+/taurocholate Cotransporting Polypeptide
rcDNA: Relaxed Circular DNA
cccDNA: Covalently Closed Circular DNA
HNF4α: Hepatocyte Nuclear Factor 4α
CREB: cAMP-Response Element Binding Protein
SIRT1: Silent Mating Type Information Regulation 2 Homolog 1
p53: Tumor Protein p53
NAD+: Nicotinamide Adenine Dinucleotide
AP-1: Activator Protein-1
PPARα: Peroxisome Proliferator-Activated Receptor Alpha
CHB: Chronic Hepatitis B
ChIP: Chromatin Immunoprecipitation
GAPDH: Glyceraldehyde-3-Phosphate Dehydrogenase
MYH6: Myosin Heavy Chain 6
PHHs: Primary Human Hepatocytes
PBMCs: Peripheral Blood Mononuclear Cells
NF-1: Nuclear Factor 1
Sp-1: Sp1 Transcription Factor
TBP: TATA-Box Binding Protein
Prox-1: Prospero Homeobox 1
HNF3α: Hepatocyte Nuclear Factor 3α
