# Supplementary material for: SIRT2 Promotes HBV Transcription and Replication by Targeting Transcription Factor p53 to Increase the Activities of HBV Enhancers and Promoters
Source: Front Microbiol. 2022 May 19;13:836446. doi: 10.3389/fmicb.2022.836446 (PMC9161175; doi:10.3389/fmicb.2022.836446)
Supplement: Supplementary file 1 [file Data_Sheet_1.DOCX]

**Supplementary Figure legends**

**Supplementary Fig. S1 The timeline for the role of SIRT2 in regulating HBV transcription and replication.** HepG2-NTCP cells were infected with of HBV for 16 h and then transduced with lentivirus expressing SIRT2 isoform 1 for 3, 5 or 7 days. (A) The overexpression efficiency of SIRT2 was determined by Western blot. (B-C) Total HBV RNAs level and HBV 3.5-kb RNA levels were measures by real-time PCR. (D) HBV core DNA level was measured by real-time PCR. (E-F) HBsAg and HBeAg levels were analyzed by ELISA. Data are shown as mean ± SD. **P*<0.05, ***P*<0.01.

**Supplementary Fig. S2 The effect of o of SIRT2 on cccDNA level. (A) The effect of overexpressing of SIRT2 before infecting the cells with HBV on cccDNA level.** HepG2-NTCP cells were transduced with lentivirus expressing SIRT2 or shSIRT2 and then infected with HBV for 5 days. The HBV cccDNA was detected by taqman probe specific real-time PCR. (B) The effect of SIRT2 on cccDNA level in HBV replication model which did not allow recycling processes. HepG2-NTCP cells were infected with HBc-deleted HBV and then transduced with lentivirus expressing SIRT2 or shSIRT2. Five days later, the HBV cccDNA was detected by taqman probe specific real-time PCR.

**Supplementary Fig. S3 The effect of SIRT2 on the acetylation level of cccDNA-bound H3/H4.** (A, B) HepG2-NTCP cells were transduced with lentivirus expressing SIRT2 or vector for 5 days. Cross-linked chromatin was immunoprecipitated with anti-AcH3 or anti-AcH4 antibody followed by real-time PCR with specific HBV cccDNA primers. The promoter of *GAPDH* and *MYH6* were used as internal controls. The ChIP results are expressed as % of input.

**Supplementary Fig. S4 The mutant p53 binding sites**. (A, B) The reported p53 binding sites in HBV EnⅠ/Xp and EnⅡ/Cp (genotype D) are indicated. The mutant p53 binding sequence was shown as bold-type letter and underlined in boxes. (C) HepG2-NTCP cells were infected with wild type HBV or p53 binding sites mutant HBV for 5 days. The recruitment of p53 to HBV EnⅠ/Xp and EnⅡ/Cp was detected by ChIP assay. The promoter of *GAPDH* and *MYH6* were used as internal controls. The ChIP results are expressed as % of input. (D) Total HBV RNAs level and HBV 3.5-kb RNA levels were measures by real-time PCR. Data are shown as mean ± SD. **P*<0.05, ***P*<0.01.

**Supplementary Fig. S5 The overexpression efficiency of SIRT2 and p53.** HBV-infected HepG2-NTCP cells were transfected with plasmids containing SIRT2 or p53. Western blot analysis confirmed the overexpression efficiency of SIRT2 and p53.

**Supplementary Fig. S6 The effect of SIRT2 on HBV transcription and replication in the absence of p53. The p53 knockout and Non-target control** HepG2-NTCP cells were infected with HBV and then transduced with lentivirus expressing SIRT2 for 5 days. (A) SIRT2 and p53 protein levels were measure by western blot. (B) Total HBV RNA and HBV 3.5-kb RNA levels were detected by real-time PCR and Northern blot. (C) HBV core DNA level was measured by real-time PCR and Southern blot.

**Supplementary Fig. S7 The effect of SIRT2 overexpression and knockdown on cell viability.** (A, B) HepG2-NTCP cells and PHHs were infected with HBV and transduced with lentivirus expressing SIRT2 or shSIRT2 for 5 days. The cell viability was analyzed by alamar blue assay.

**Supplementary Fig. S8 The cellular localization of SIRT2.** (A, B) HepG2-NTCP cells were infected with HBV particles. Real-time PCR determined the HBV core DNA level in HBV-infected HepG2-NTCP cells (A). Western blot analyzed SIRT2 level in nuclear and cytoplasmic fractions of HBV-infected and HBV-uninfected HepG2-NTCP cells (B).

**Supplementary Fig. S9 The effect of SIRT2 on the H3/H4 acetylation binding to p53 promoter.** (A, B) HepG2-NTCP cells were transduced with lentivirus expressing SIRT2 for 3 days. the H3/H4 acetylation level of p53 promoter was analyzed by ChIP assay. The promoter of *GAPDH* and *MYH6* were used as internal controls. The ChIP results are expressed as % of input.
