# Supplementary material for: SIRT2 Promotes HBV Transcription and Replication by Targeting Transcription Factor p53 to Increase the Activities of HBV Enhancers and Promoters
Source: Front Microbiol. 2022 May 19;13:836446. doi: 10.3389/fmicb.2022.836446 (PMC9161175; doi:10.3389/fmicb.2022.836446)
Supplement: Supplementary file 2 [file Table_1.DOCX]

Table 1 Clinical characteristic of heathy volunteers and patients with CHB.

| Characteristics | Healthy (25) | CHB (25) | **P* (<0.05) |
| --- | --- | --- | --- |
| Age(years) | 30.84±9.21 | 29.48±7.66 | 0.5730(ns) |
| Gender(M/F) | 10/15 | 14/11 | 0.396(ns) |
| ALT(U/L) | 17.88±12.74 | 85.08±56.17 | 0.0001(***) |
| AST(U/L) | 17.96±5.97 | 75±54.95 | 0.0001(***) |
| ALB(g/L) | 43.26±6.94 | 42.01±4.34 | 0.0297(*) |
| GLB(g/L) | 28.31±2.93 | 31.06±3.54 | 0.0009(***) |
| TBIL(μmol/L) | 14.05±6.93 | 20.75±21.88 | 0.1903(ns) |
| AFP(ng/mL) | 2.52±2.62 | 4.04±8.11 | 0.8513(ns) |

All values are expressed as the mean±Standard deviation.

Abbreviations: F, Female; M, Male; ALT, alanine aminotransferase; AST, aspartate aminotransferase; ALB, Albumin; GLB, Globulin; TBIL, Total Bilirubin; AFP, Alpha Fetoprotein. (* *P*<0.05, *** *P*<0.001, n.s., not significant)
