# Supplementary material for: SIRT2 Promotes HBV Transcription and Replication by Targeting Transcription Factor p53 to Increase the Activities of HBV Enhancers and Promoters
Source: Front Microbiol. 2022 May 19;13:836446. doi: 10.3389/fmicb.2022.836446 (PMC9161175; doi:10.3389/fmicb.2022.836446)
Supplement: Supplementary file 3 [file Table_2.DOCX]

**Supplementary Table 1**

| HBV core DNA forward | 5’-CCTAGTAGTCAGTTATGTCAAC-3’ |
| --- | --- |
| HBV core DNA reverse | 5’-TCTATAAGCTGGAGGAGTGCGA-3’ |
| total HBV RNAs forward | 5’- ACCGACCTTGAGGCATACTT-3’ |
| total HBV RNAs reverse | 5’- GCCTACAGCCTCCTAGTACA-3’ |
| HBV 3.5-kb RNA forward | 5’- GCCTTAGAGTCTCCTGAGCA-3’ |
| HBV 3.5-kb RNA reverse | 5’- GAGGGAGTTCTTCTTCTAGG-3’ |
| β-actin forward | 5’-CTCTTCCAGCCTTCCTTCCT-3’ |
| β-actin reverse | 5’- AGCACTGTGTTGGCGTACAG-3’ |
| cccDNA forward | 5’- CTCCCCGTCTGTGCCTTCT-3’ |
| cccDNA reverse | 5’-CCCCAAAGCCACCCAAG-3’ |
| cccDNA probe | 5’-TTCATCCTGCTGCTATGCCTGATCTTCTTG-3’ |
| HBV EnⅠ/Xp ChIP forward | 5’-GTGGTTATCCTGCGTTGATG-3’ |
| HBV EnⅠ/Xp ChIP reverse | 5’-CGTCAGCAAACACTTGGCAC-3’ |
| HBV EnⅡ/Cp ChIP forward | 5’-CCACCAAATATTGCCCAAGG-3’ |
| HBV EnⅡ/Cp ChIP reverse | 5’-AGCCTCCTAGTACAAAGACC-3’ |
| p53 ChIP forward | 5’ GTTAGTATCTACGGCACCAG-3’ |
| p53 ChIP reverse | 5’-GGACAGTCGCCATGACAAGT-3’ |
| GAPDH ChIP forward | 5’-TACTAGCGGTTTTACGGGCG-3’ |
| GAPDH ChIP reverse | 5’-TCGAA AGGAGGAGCAGAGAGCGA-3’ |
| MYH6 ChIP forward | 5’-AGAAGCTGCGCTCAGACCTGTCTCG-3’ |
| MYH6 ChIP reverse | 5’-TCCAGGTCCCGCCGCATCTT-3’ |
| SIRT2 forward | 5’- CCGGCCTCTATGACAACCTA-3’ |
| SIRT2 reverse | 5’- GGAGTAGCCCCTTGTCCTTC-3’ |
| shSIRT2-1 | 5’- GCCAACCATCTGTCACTACTT-3’ |
| shSIRT2-2 | 5’- GCTAAGCTGGATGAAAGAGAA-3’ |
| shCont | 5′-GCAACAAGATGAAGAGCACCAA-3′ |
| PPARα forward | 5’-TGGAGCATTGAACATCGAAT-3’ |
| PPARα reverse | 5’-GGTCGCACTTGTCATACACC-3’ |
| Sp1 forward | 5’-CTGAAGCTGGGTAGCCTATTG-3’ |
| Sp1 reverse | 5’-CTACTGCTGCGACCTTTCTT-3’. |
| TBP forward | 5’-GCTCTCATGTACCCTTGCCT-3’ |
| TBP reverse | 5’-GCACTTACAGAAGGGCATCA-3’ |
| c-Jun forward | 5’-GAGCTGGAGCGCCTGATAAT-3’ |
| c-Jun reverse | 5’- CCCTCCTGCTCATCTGTCAC-3’ |
| Prox1 forward | 5’-AACTAGGGATACCACGAGTC-3’ |
| Prox1 reverse | 5’-CTTCACTATCCAGCTTGCAG-3’ |
| CREB forward | 5’-ACCTGCCATCACCACTGTAA-3’ |
| CREB reverse | 5’-GTATTGCTCCTCCCTGGGTA-3’ |
| p53 forward | 5’-AACAACACCAGCTCCTCTCC-3’ |
| p53 reverse | 5’-CTCATTCAGCTCTCGGAACA-3’ |
| HNF3α forward | 5’-CAGCAAACAAAACCACACAAACC-3’ |
| HNF3α reverse | 5’-ACACTTGTGGATCATTAAACTTCGC-3’ |
| HNF3β forward | 5’-GTTGTTGTTGTTCTCCTCCATTGC-3’ |
| HNF3β reverse | 5’-AACTACATGGTTTTACACCGAGTCAC-3’ |
| C/EBP α forward | 5’-CGAGCCAGGACTAGGAGATT-3’ |
| C/EBP α reverse | 5’-CCTCATCTTAGACGCACCAA-3’ |
| C/EBP β forward | 5’-CTGGAGACGCAGCACAAG-3’ |
| C/EBP β reverse | 5’-ACAGCTGCTCCACCTTCTTC-3’ |
| HNF1α forward | 5’-CTCATCACCGACACCACCAA-3’ |
| HNF1α reverse | 5’-GAAGACCTGGGGGAGCAGA-3’ |
| HNF4α forward | 5’-GCCTACCTCAAAGCCATCAT-3’ |
| HNF4α reverse | 5’-CGGTCGTTGATGTAGTCCTC-3’ |
| TR4 forward | 5’-TTGTGAAGGTTGCAAAGGTT-3’ |
| TR4 reverse | 5’-TTCATGCCCATCTCTAAGCA-3’ |
| COUP-TF forward | 5’-ACTGCTACCTGTCCGGCTAC-3’ |
| COUP-TF reverse | 5’-TCGATGCCCATAATGTTGTT-3’ |
| PGC-1α forward | 5’-CTGTGGATGAAGACGGATTG-3’ |
| PGC-1α reverse | 5’-AGCCTCATTGTCAGTGGTCA-3’ |
| p65 forward | 5’-ATAGAAGAGCAGCGTGGGGA-3’ |
| p65 reverse | 5’-GATCTTGAGCTCGGCAGTGT-3’ |
| TR2 forward | 5’-CTCCAGACCAAGGACCAAAT-3’ |
| TR2 reverse | 5’-AAATCCTTTGCAGCCTTCAC-3’ |
| RXRα forward | 5’-GGACATGCAGATGGACAAGA-3’ |
| RXRα reverse | 5’-CCCTTGGAGTCAGGGTTAAA-3’ |
| OCT1 forward | 5’-TGCAGACAGGTTTGGCCGT-3’ |
| OCT1 reverse | 5’-GCCCGAGCCAACAAATTCTGTGAT-3’ |
