# Supplementary figures and images for: SIRT2 Promotes HBV Transcription and Replication by Targeting Transcription Factor p53 to Increase the Activities of HBV Enhancers and Promoters
Source: Front Microbiol. 2022 May 19;13:836446. doi: 10.3389/fmicb.2022.836446 (PMC9161175; doi:10.3389/fmicb.2022.836446)

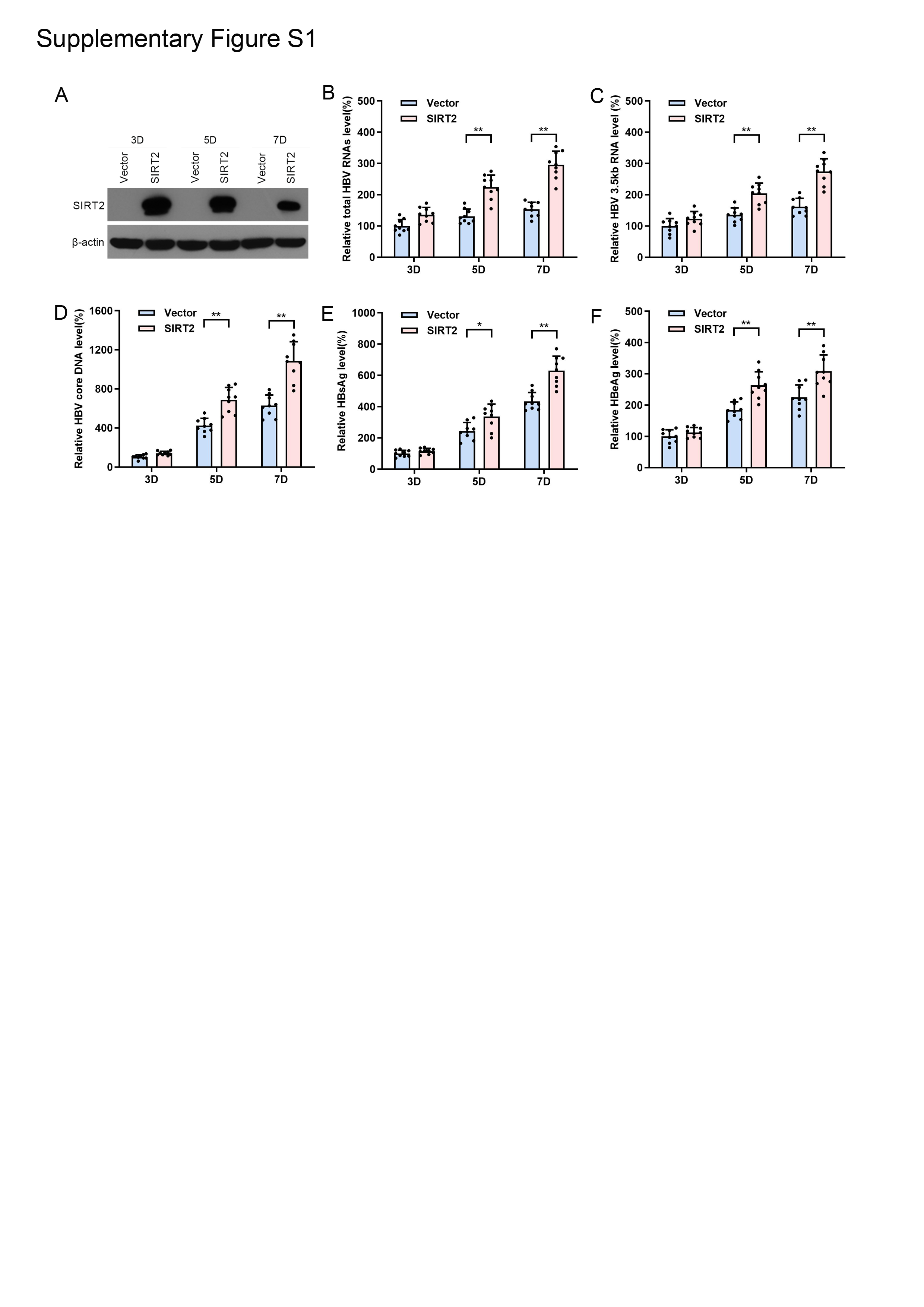

Supplement: Supplementary file 4 [file Image_1.TIF]

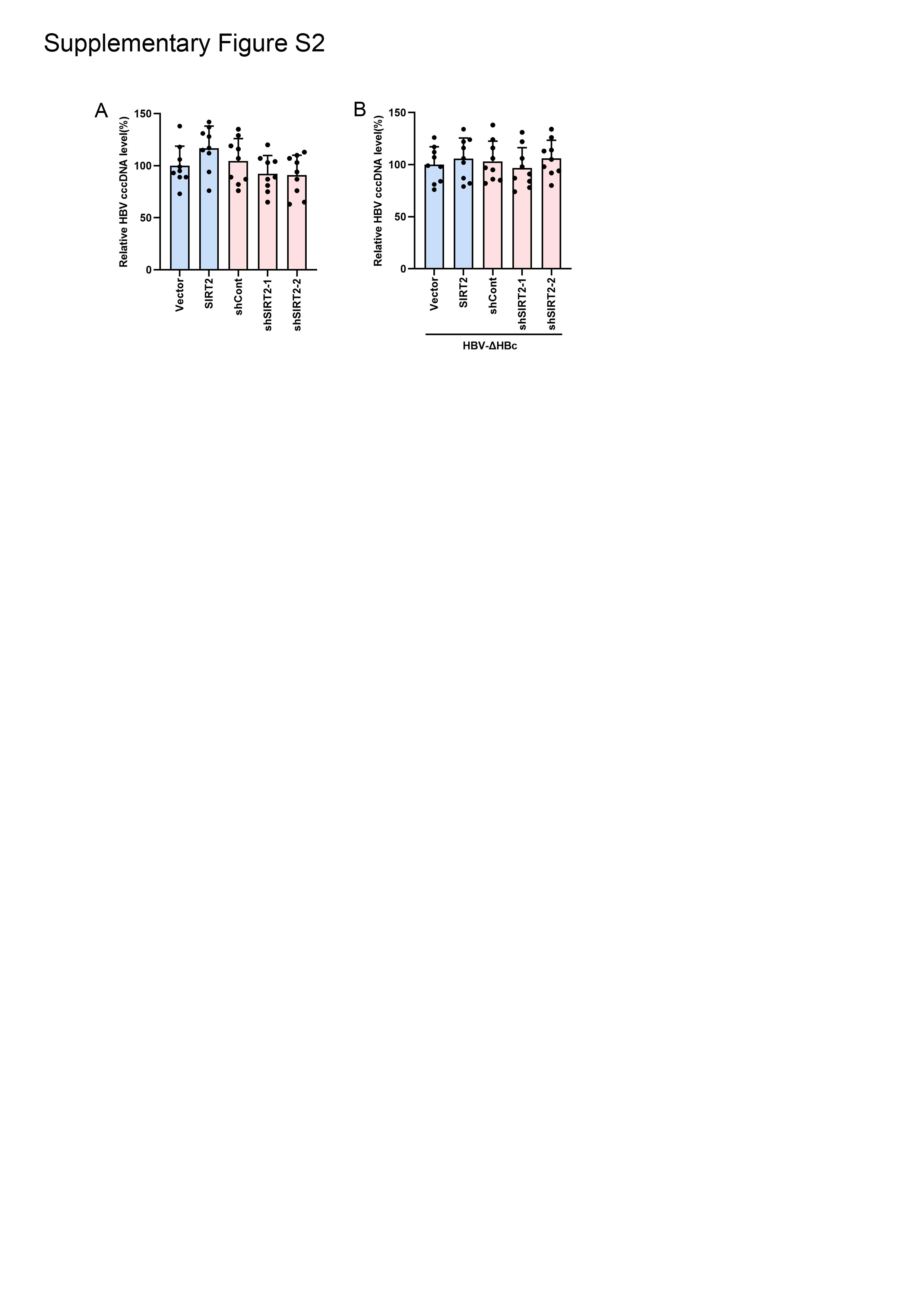

Supplement: Supplementary file 5 [file Image_2.TIF]

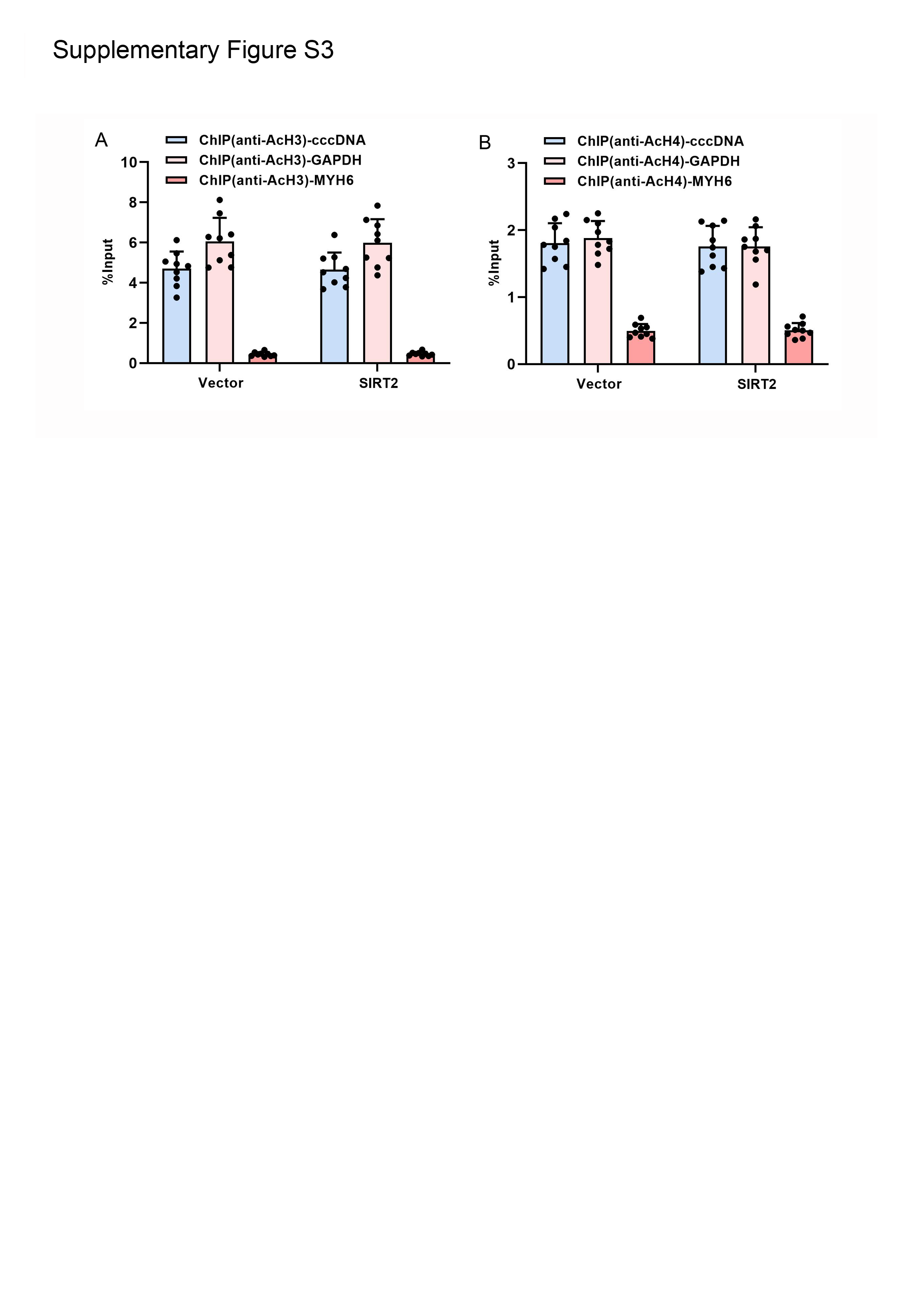

Supplement: Supplementary file 6 [file Image_3.TIF]

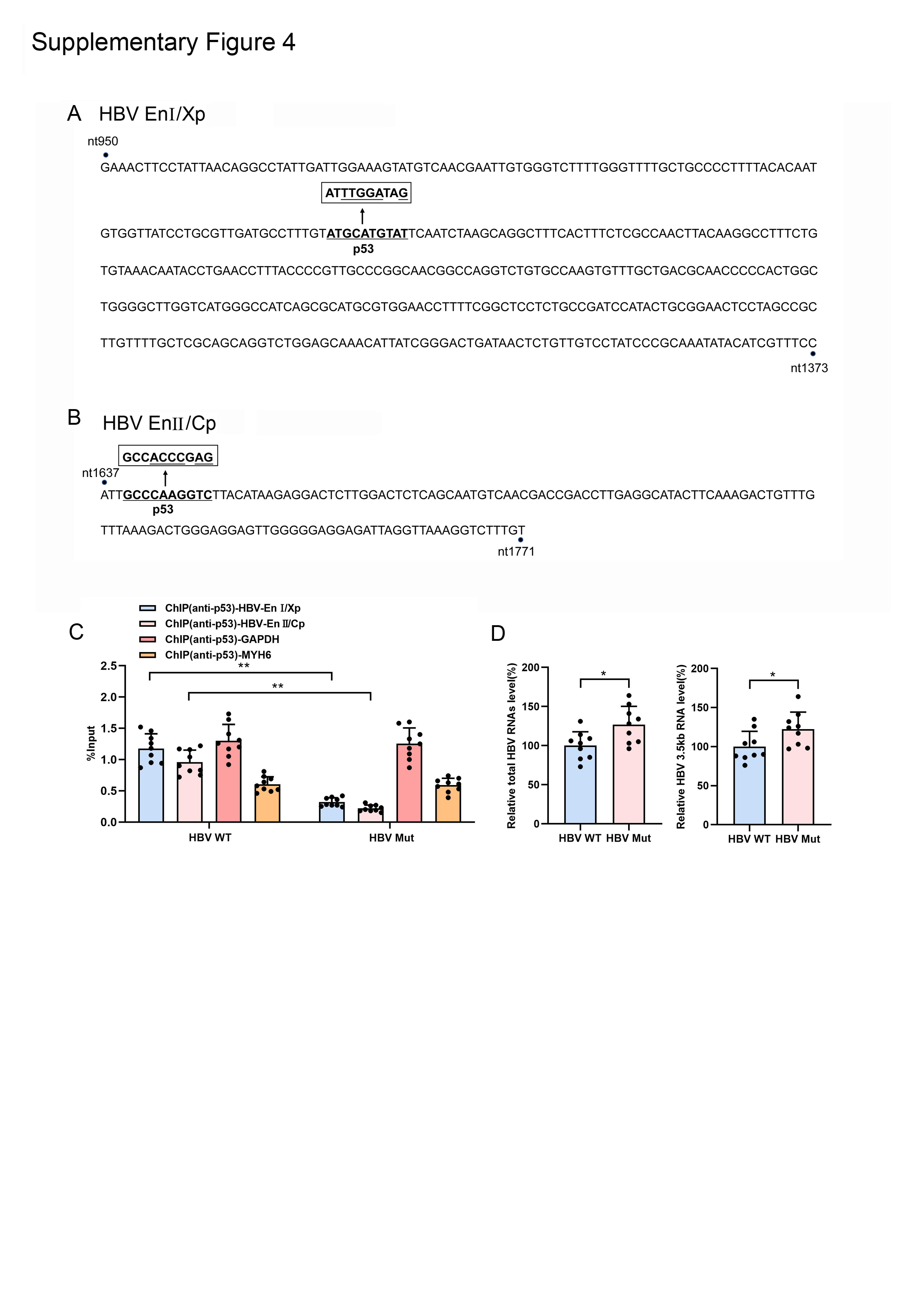

Supplement: Supplementary file 7 [file Image_4.TIF]

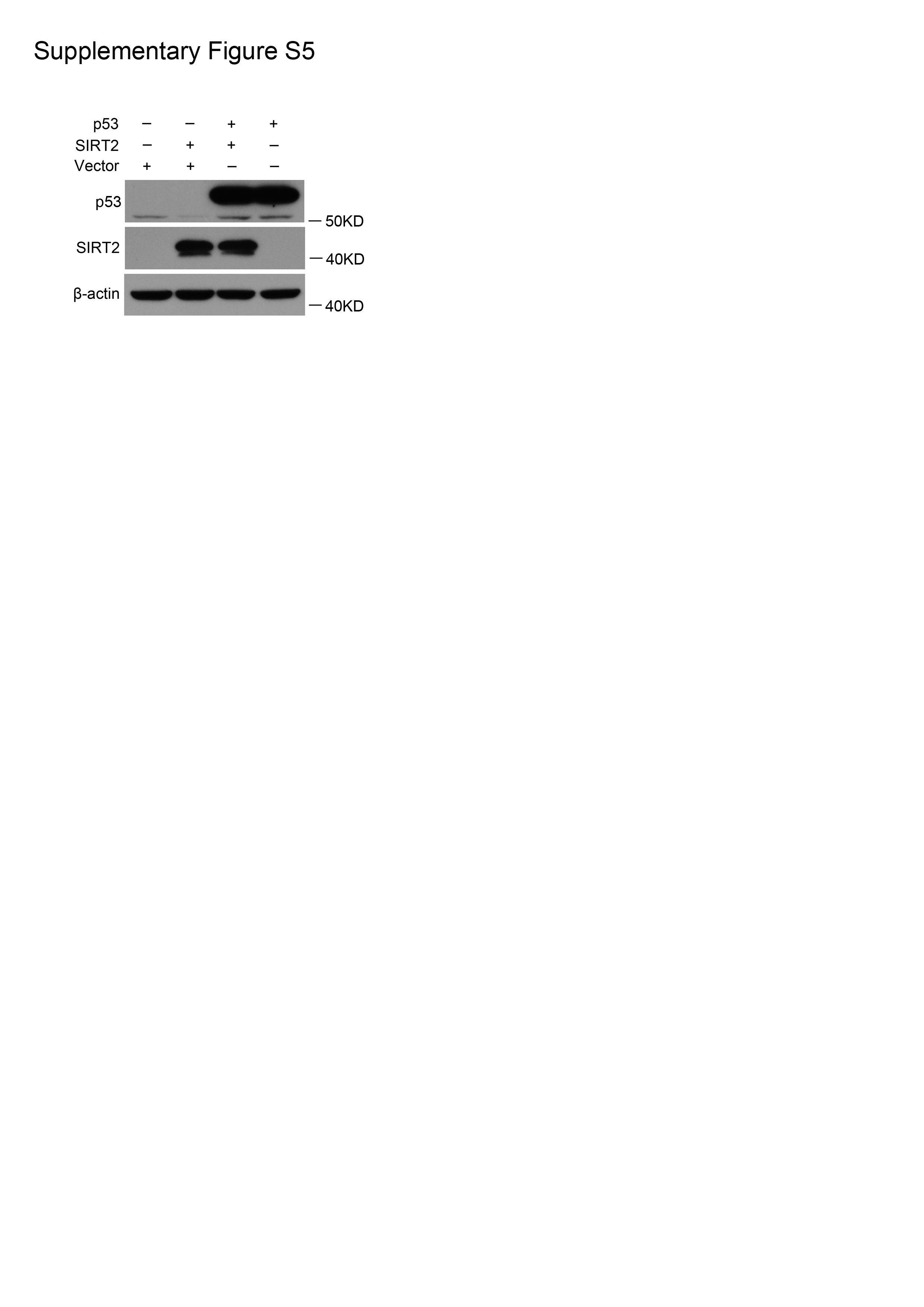

Supplement: Supplementary file 8 [file Image_5.TIF]

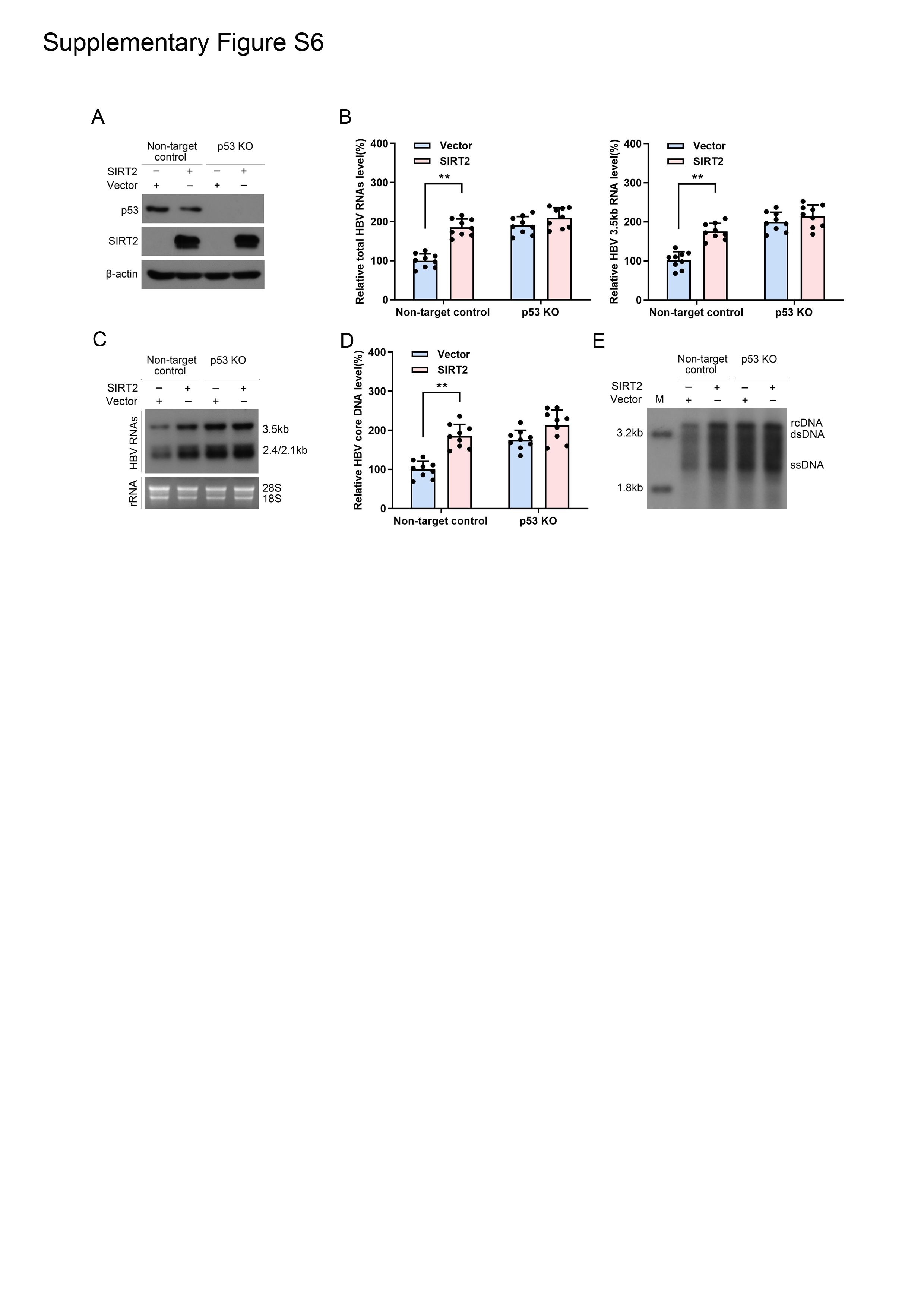

Supplement: Supplementary file 9 [file Image_6.TIF]

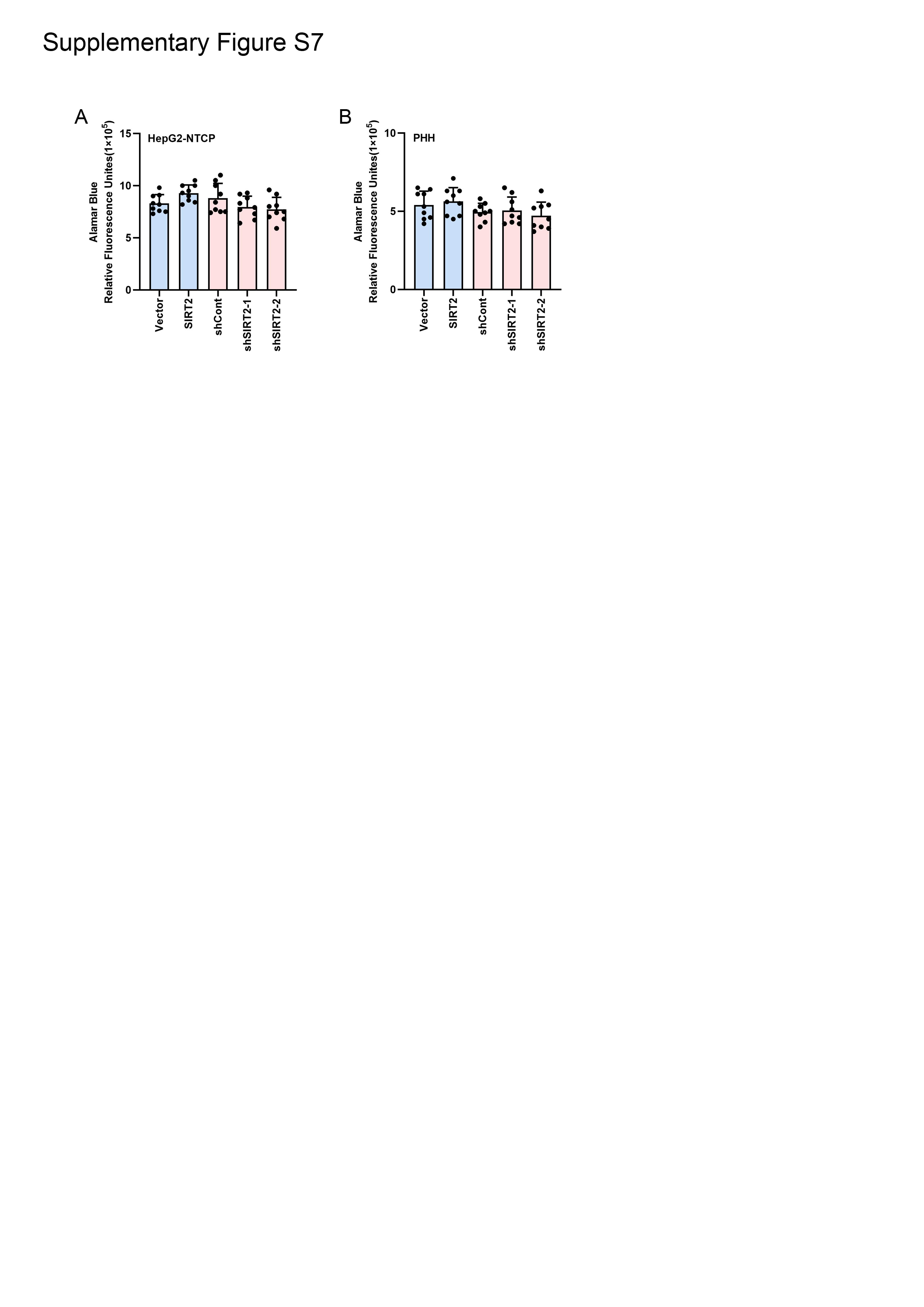

Supplement: Supplementary file 10 [file Image_7.TIF]

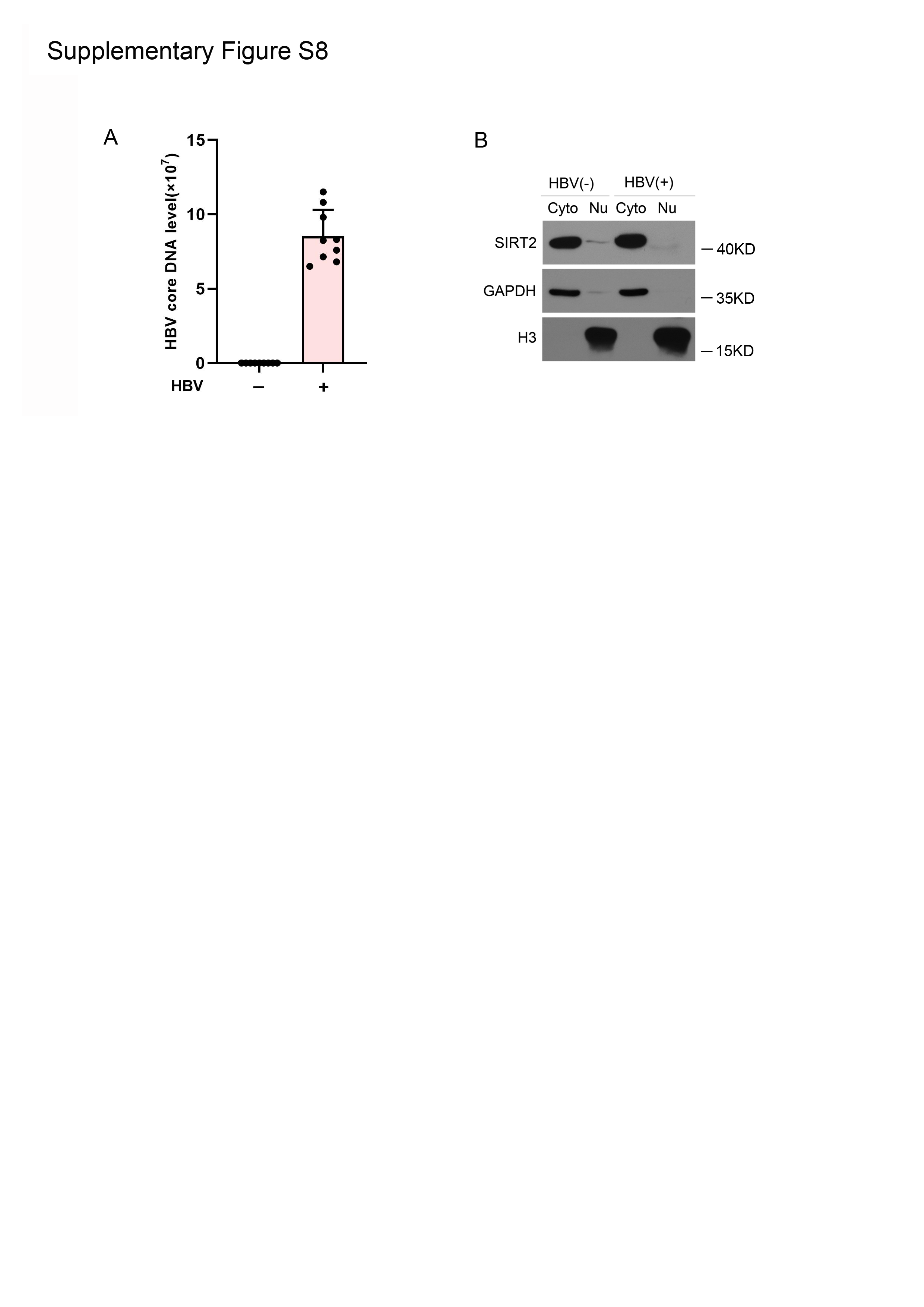

Supplement: Supplementary file 11 [file Image_8.TIF]

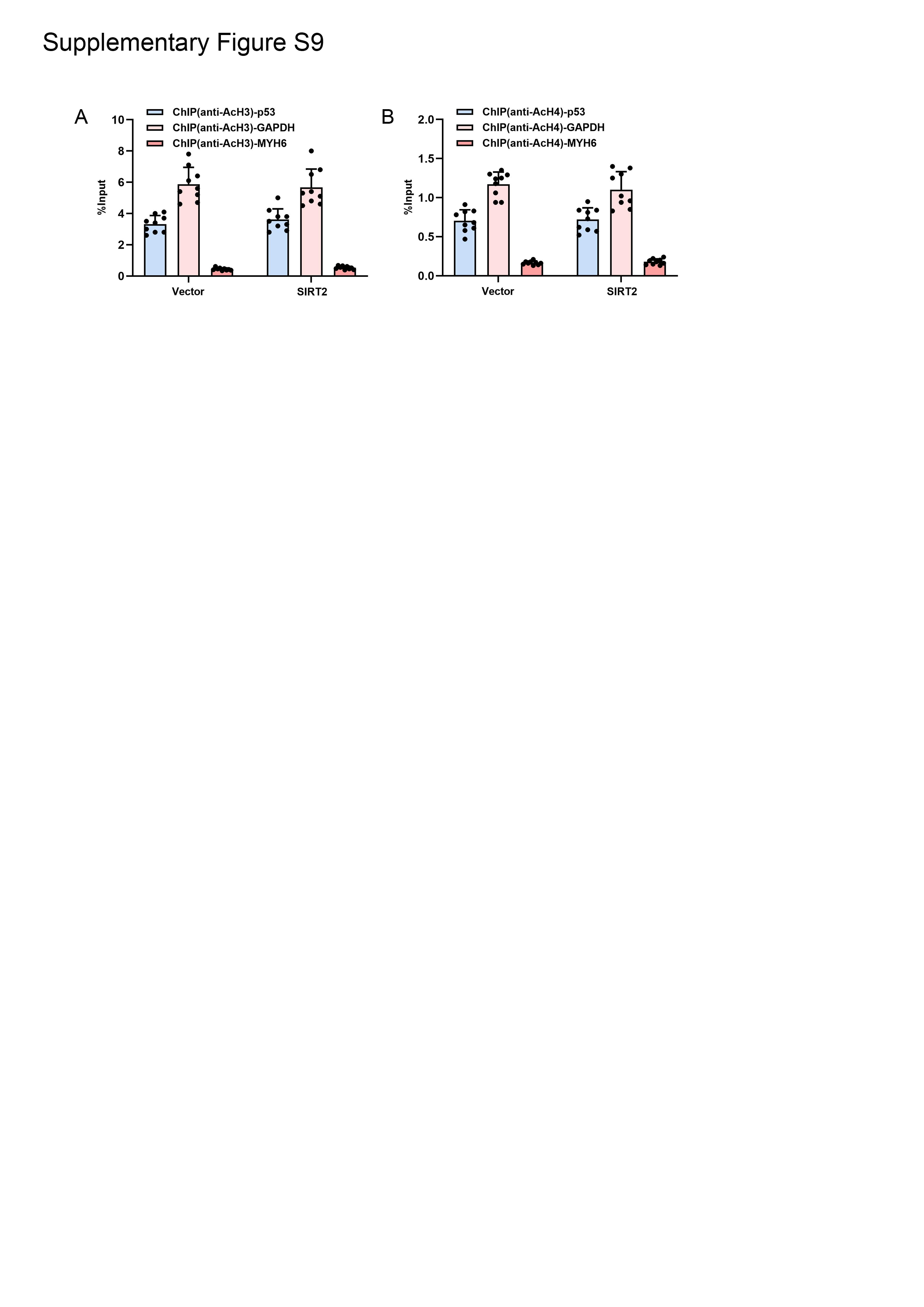

Supplement: Supplementary file 12 [file Image_9.TIF]
